# Coherent detector for the non-separability measurement of vectorial structured light

**DOI:** 10.1038/s41377-025-02035-1

**Published:** 2025-09-26

**Authors:** Yize Liang, Shuai Cao, Lixian Liu, Fei Liu, Xukun Yin, Pei Lv, Yiming Zhang, Yunrui Zou, Liang Fang, Shuang Zheng, Zhenyu Wan, Teli Xi, Xiaopeng Shao, Jian Wang

**Affiliations:** 1 School of Optoelectronic Engineering, Xidian University, Xi’an, 710071 China; 2 Wuhan National Laboratory for Optoelectronics and School of Optical and Electronic Information, Huazhong University of Science and Technology, Wuhan, 430074 Hubei China; 3 Optics Valley Laboratory, Wuhan, 430074 Hubei China; 4 School of Physics and Electronics, Hunan University, Changsha, 410082 China; 5 Xi’an Institute of Optics and Precision Mechanics, CAS, Xi’an, 710119 China; 6 Hubei Optical Fundamental Research Center, Wuhan, 430074 China

## Abstract

Atmospheric turbulence distorts the complex wavefront of light in free-space optical communication systems, leading to bit errors and even communication interruptions. Recently, it is found that the non-separability of vectorial structured light remains invariant when transmitting through atmospheric turbulence. This discovery offers a potential solution for turbulence-resilient communications—encoding based on the non-separability of vectorial structured light. To achieve such turbulence-resilient communications, efficient detection of the non-separability of vectorial structured light is essential, which acts as the receivers of such communication systems. So far, traditional non-separability detection schemes usually rely on bulky SLMs or DMDs, facing inherent trade-offs between single-shot capability and system compactness. In addition, the detection of mode-resolved non-separability contributions of vectorial superposition states has not yet been accomplished. Here, we propose and experimentally demonstrate a coherent detector to characterize the non-separability of vectorial structured light based on off-axis digital holography, which overcomes the limitations of traditional approaches by digitally decomposing spatial modes. Our approach may pave the way for turbulence-resilient optical communications based on non-separability coding methods and bring new insights into non-separability measurement.

## Introduction

Free-space optics (FSO), which is also known as wireless optical communications, has attracted tremendous attention due to its large transmission capacity, license-free frequency spectrum, and immunity against eavesdropping^1–3^. In recent years, so-called structured light^4–9^ offers a new spatial degree of freedom^10–13^ for FSO to improve the capacity per transmission channel^14–17^. However, atmospheric turbulence in free-space links distorts the complex wavefront of structured light, inducing modal crosstalk between different structured light modes^18–20^. Such modal crosstalk disables the long-haul transmission of structured light in free space^21,22^. Thus, lots of effort have been devoted to searching for an approach which can efficiently transmit information loaded on structured light to the receiver through atmospheric turbulence^23–28^.

Remarkably, in 2022, it is found that the non-separability^29–33^ of vectorial structured light remains invariant in unitary and one-sided channels (e.g., atmospheric turbulence)^34,35^. This discovery provides a convincing and new solution for turbulence-resilient communications based on structured light—packing information into the non-separability of vectorial structured light^36^. Similar to loading 1 and 0 on different light pulse intensities in on-off keying (OOK) intensity modulation, this new solution is capable of encoding 1 and 0 onto vectorial structured light with different non-separability. Furthermore, the new solution may be more efficient than OOK modulation, since the whole range of non-separability from 0 to 1 can be defined as a N-dimensional alphabet.

To establish such an efficient free-space optical communication link based on non-separability coding method, precise and fast acquisition of the non-separability of the received vectorial structured light is urgent required. It functions similarly to photodetectors in traditional optical communications, which determines the capacity and bit-error rate (BER) performance of a communication system. To date, approaches to detecting the non-separability of received vectorial structured light can be divided into two categories: basis-independent methods and basis-dependent approaches. The basis-independent methods measure the Stokes parameters of vectorial structured light field to characterize its non-separability. While the basis-dependent approaches usually obey such a tomography principle: sorting the spatial terms and polarization terms of vectorial structured light based on polarizing optics and demodulated holograms loaded onto spatial light modulators (SLMs) or digital micromirror devices (DMDs), thus the intensity coefficients of different basic states can be directly measured to determine the non-separability^29,37–41^. For these two kinds of non-separability detection methods, since the non-separability is calculated by a direct detection of the intensity of light beams (i.e., measuring the Stokes parameters based on intensity profiles of light passing through polarizing optics or detecting the intensity of demodulated light), these traditional approaches act like direct detectors in intensity modulation and direct detection (IM-DD) communications. To summarize, these approaches usually feature some of these three drawbacks: i) they are limited by the performance and high cost of SLMs and DMDs; ii) single-shot detection and the simplicity of detection system are usually incompatible, since characterizations of the intensity coefficients of different basic states are accomplished through multiple demodulation measurements in the time domain or simultaneous measurements at different positions in space domain; iii) detection of the non-separability of vectorial structured light superposition states has not been accomplished. These drawbacks are obstacles to the practical use of non-separability coding communications.

Here, we propose and experimentally demonstrate a coherent detector to efficiently detect the non-separability of vectorial structured light, which addresses the mentioned three drawbacks. The coherent detector splits two circularly polarized components of vectorial structured light and reconstructs their complex phase front based on off-axis digital holography. Calculating the overlapping degree (i.e., inner product) between ideal complex phasefront of different basic states and the retrieved complex phasefront enables characterizing the intensity coefficients of different basic states to obtain the non-separability. Like coherent receivers that achieve decoding through interferometric phase measurement in coherent optical communications, the non-separability coherent detector proposed in this paper characterize the non-separability based on the acquired phasefront of light beams through interference. Compared to conventional methods for determining the non-separability of vectorial structured light, this non-separability coherent detector forgoes demodulating basic spatial states using bulky SLMs or DMDs yet instead sorting them in digital processing. Hence, the proposed approach breaks the limitation of SLMs and DMDs, enabling single-shot detection of the non-separability of vectorial structured light. In addition, single-shot detection of the non-separability contributions of vectorial structured light superposition states is also demonstrated. Thus, our approach may pave the way for turbulence-resilient optical communications based on non-separability coding methods. Moreover, considering that lots of efforts have been devoted to creating more complex non-separable states^42–47^, our technique may provide an efficient tool for characterizing the non-separability of such complex states.

## Results

### Concept and experimental setup

The non-separability of a vectorial structured light beam, also named as vectorness or vector quality factor (VQF), describes how “vector” the beam is. To start with, here we recall the expression of a vectorial structured light given by the superposition of optical vortex (OV) beams with non-separable orbital angular momenta (OAM) and spin angular momenta (SAM),

1 $$V(r,\theta )=\,\cos \,\theta {E}_{OV}(\pm l){{\bf{e}}}_{{\bf{L}}}+{e}^{i2\alpha }\,\sin \,\theta {E}_{OV}({\rm{\mp}}l){{\bf{e}}}_{{\bf{R}}}$$

where ${E}_{OV}(l)$ and ${E}_{OV}(-l)$ represent OV beams with topological charges of *l* or -*l*, which can be written as ${e}^{il\varphi }$ or ${e}^{-il\varphi }$, respectively. ${{\bf{e}}}_{{\bf{L}}}$ and ${{\bf{e}}}_{{\bf{R}}}$ correspond to left-circularly polarized (LCP) and right-circularly polarized (RCP) states, $2\alpha$ describes the relative phase difference of two OV beam components. The parameter $\theta$ determines how “vector” the beam is. For instance, the vectorial structured light beam defined by Eq. (1) will be a pure vector beam or a pure scalar beam when $\theta =(2n+1)\pi /4$ or $\theta =n\pi /2$ (n = 0,1,2,…), respectively. The non-separability of a vectorial structured light beam can be defined as^37,39^,

2 $${\text{Non}}-{\text{separability}}={\text{Re}}(C)={\text{Re}}(\sqrt{1-{s}^{2}})=|{\text{sin}}(2\theta )|$$

where the concurrence *C*^48,49^ describes the degree of entanglement, *s* is the length of the Bloch vector and can be defined as $s=\sqrt{({\sum }_{i=1}^{3}{\langle {\sigma }_{i}\rangle }^{2})}$. Here, ${\sigma }_{1}$, ${\sigma }_{2}$ and ${\sigma }_{3}$ are the expectation values of the Pauli operators. They can be calculated through the power coefficients of six different basic states by using the following formula,

3 $$\left\{\begin{array}{l}\langle {\sigma }_{1}\rangle =({P}_{3L}+{P}_{3R})-({P}_{4L}+{P}_{4R})\\ \langle {\sigma }_{2}\rangle =({P}_{5L}+{P}_{5R})-({P}_{6L}+{P}_{6R})\\ \langle {\sigma }_{3}\rangle =({P}_{1L}+{P}_{1R})-({P}_{2L}+{P}_{2R})\end{array}\right.$$

where ${P}_{{i}L}$ and ${P}_{{i}R}$ (*i* = 1,…,6) are the power coefficients of the *i*-th basic state in LCP channel or RCP channel, respectively. The six basic states contain two pure OV modes ${E}_{OV}(l)$, ${E}_{OV}(-l)$.and four superposition states ${E}_{OV}(l)+{e}^{i\beta }{E}_{OV}(-l)$ which can be defined by the inter-modal angle $\beta =0,\pi ,\pi /2,3\pi /2$. In traditional basis-dependent direct detection approaches, ${P}_{{i}L}$ and ${P}_{{i}R}$ can be characterized by performing a full-state tomography of the vectorial structured light beam. This full-state tomography acquires the intensity coefficients of six different basic states through multiple demodulation measurements in time domain or demultiplexing light beams to different positions in space domain. For example, the full-state tomography can be achieved by demodulating light beams with six different holograms and measuring the on-axis beam intensity. For these conventional approaches, bulky SLMs or DMDs are required to demodulate light beams in different polarization channels. Hence, they are limited by the performance and high cost of SLMs and DMDs, and the single-shot detection and the simplicity of detection system are usually incompatible. In addition, detection of the non-separability of vectorial structured light superposition states has not been accomplished by utilizing these conventional direct detection methods.

**Fig. 1:**
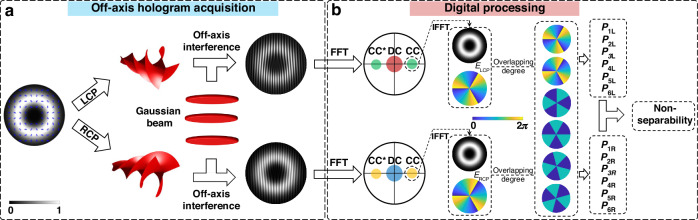
Concept of the proposed coherent detector for the non-separability measurement of vectorial structured light. **a** Concept of the off-axis hologram acquisition process. **b** Concept of the digital processing process. FFT fast Fourier transform, IFFT inverse fast Fourier transform, CC cross-correlation, DC autocorrelation, LCP left-circularly polarized, RCP right-circularly polarized

Here, we propose and experimentally demonstrate a coherent detector to efficiently detect the non-separability of vectorial structured light, which overcomes the mentioned limitations of conventional direct detection methods. Figure 1 illustrates the concept of the proposed coherent detector for the non-separability measurement, including an off-axis hologram acquisition process and a digital processing part. As shown in Fig. 1a, a vectorial structured light beam is polarization split into LCP beam and RCP beam by polarizing optics, thus the two polarized components in Eq. (1) are separated. Then a Gaussian beam with a large beam waist radius off-axis interferes with the two signal beams in LCP channel and RCP channel. As a result, off-axis interference holograms with linear fringes are captured by a charge-coupled device (CCD). The direction of the linear fringes in off-axis interference holograms depends on transmission direction difference between two interference beams, while the period of the linear fringes is determined by the value of their off-axis angle.

Once the off-axis interference holograms in LCP channel and RCP channel are obtained, they will undergo a digital processing procedure to determine the final non-separability, as indicated in Fig. 1b. Firstly, fast Fourier transform (FFT) is performed on the recorded off-axis interference holograms to acquire their spatial-frequency (SF) domain. In the SF domain, cross-correlation (CC) terms are separated from autocorrelation (DC) terms^50–52^. The CC terms contain the phasefront difference information between signal beams and reference beams. Since the large Gaussian beam tends to possess a flat phasefront, applying inverse fast Fourier transform (IFFT) on the CC terms enables single-shot detection of the complex wavefronts of signal beams. Note that the complex wavefronts including intensity profiles and phasefront of signal beams are retrieved, power coefficients of different basic states can be characterized by calculating the overlapping degree between ideal complex wavefronts of basic states and the retrieved complex wavefronts. The overlapping degree between different modes, which is also known as their inner product, can be described as follows,

4 $$\left\{\begin{array}{l}{P}_{iL}={|\iint {E}_{ret,L}(x,y){E}_{i,L}^{\ast }(x,y)dxdy|}^{2}\\ {P}_{iR}={|\iint {E}_{ret,R}(x,y){E}_{i,R}^{\ast }(x,y)dxdy|}^{2}\end{array}\right.$$

where ${P}_{iL}$ and ${P}_{iR}$ denote the intensity coefficient of the *i*-th basic mode in LCP channel or RCP channel. ${E}_{ret,L}(x,y)$ and ${E}_{ret,R}(x,y)$ represent the experimentally retrieved complex wavefronts in LCP channel or RCP channel, respectively. ${E}_{i,L}^{\ast }(x,y)$ and ${E}_{i,R}^{\ast }(x,y)$ correspond to the conjugation of the complex wavefronts of *i*-th basic mode in LCP channel or RCP channel. For example, the first and second basic mode can be expressed as ${E}_{OV}(l)={e}^{il\varphi }$ and ${E}_{OV}(-l)={e}^{-il\varphi }$, where $\varphi$ is the azimuthal coordinate.

Based on Eq. (5), all the ${P}_{iL}$ and ${P}_{iR}$ are determined to calculate the final non-separability. It is worth mentioning that ${P}_{iL}$ and ${P}_{iR}$ are normalized due to the sum of the power coefficients of a set of complete orthogonal mode basis is 1. For instance, ${E}_{OV}(l)$ beams with LCP and RCP polarization, and ${E}_{OV}(l)$ beams with LCP and RCP polarization provide a complete and orthogonal mode basis, resulting in ${P}_{1L}+{P}_{1R}+{P}_{2L}+{P}_{2R}=1$. Therefore, ${P}_{iL}$ and ${P}_{iR}$ can be decided to finally compute the non-separability of tested vectorial structured light beam based on Eq. (2) and Eq. (3).

**Fig. 2:**
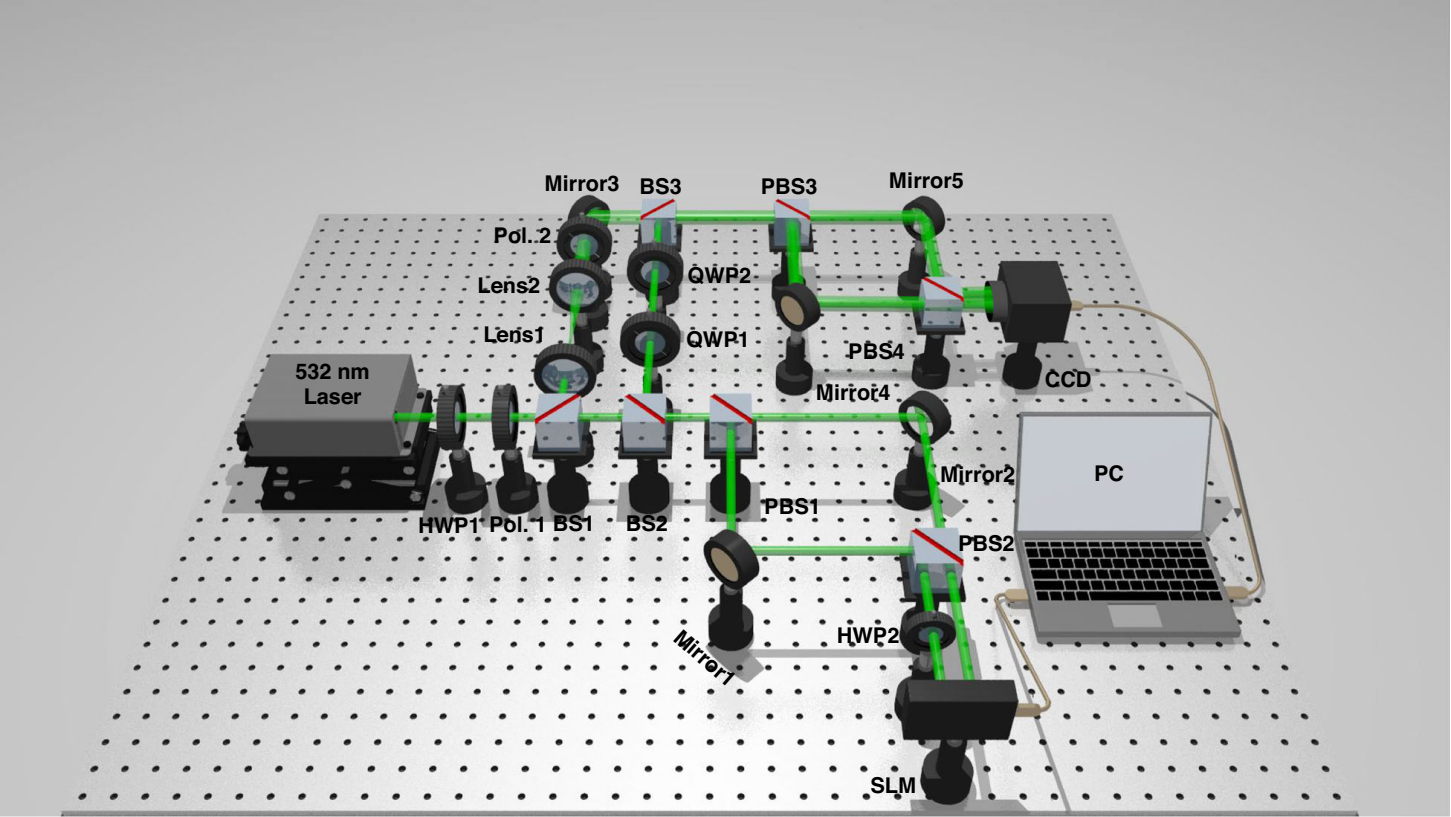
Experimental setup for the generation and coherent detection of vectorial structured light with different non-separability. Pol. polarizer, BS beam splitter, PBS polarization beam splitter, HWP half-wave plate, SLM spatial light modulator, QWP quarter-wave plate, CCD charge-coupled device, PC personal computer

To demonstrate the proposed coherent detector for the non-separability measurement of vectorial structured light, a proof-of-concept experiment is carried out. Figure 2 displays the proof-of-concept experimental setup for the generation and coherent detection of vectorial structured light with different non-separability. A 532 nm laser (Laser Quantum, torus 532) which emits 532 nm coherent Gaussian beam is chosen as the light source of our experiment. The free-space Gaussian beam propagates along the green line in Fig. 2. A polarizer (Pol. 1) is utilized to control the polarization of the transmitting light. A half-wave plate (HWP1) is applied to control the intensity of Gaussian beam after Pol. 1. Then the linearly polarized Gaussian beam is 50:50 split into two parts at a beam splitter (BS1, LBTEK BS1455-A). The straight transmission path is the signal arm that will be modulated into a vectorial structured light beam, while the reflection path is the reference arm for the off-axis interference.

Here we firstly introduce the transmission path and process to convert the linearly-polarized Gaussian signal beam into a vectorial structured light beam. The Gaussian beam straightly propagates through BS2 with a 3 dB loss, and is split into two orthogonal linear polarization components by utilizing a polarization beam splitter (PBS1). The *x*-directional polarization component straightly passes through PBS1, while the y-directional polarization component reflects at PBS1. Then the two polarization components of light transmit through a loop composed of PBS1, mirror1, mirror2 and PBS2. The two polarization components parallelly output the loop at PBS2 with a slight distance. Considering that the applied SLM is a polarization-sensitive device, HWP2 is inserted into the path of *y*-directional polarization component to align its polarization to the optimal working direction of SLM. Hence, the two polarization components of Gaussian beams can be converted into OV beams with opposite topological charges by loading two different phase holograms onto different positions on the SLM. The two modulated OV beams reflect at SLM and then re-enter the loop. They are combined and output from the left side of PBS1. Then the combined light beam reflects at BS2 with a 3-dB loss and arrives at a quarter-wave plate (QWP1). The QWP1 converts *x*-directional polarization and *y*-directional polarization into LCP state and RCP state, respectively. Thus, a composition of LCP OV beams and RCP OV beams with opposite topological charges can be obtained after QWP1, which forms the vectorial light field in Eq. (1).

Then the experimental transmission path and process for measuring the non-separability of vectorial structured light beams are described. The generated vectorial structured light beam is converted into a combination of *x*-directional polarized OV beam and *y*-directional polarized OV beam by utilizing QWP2. Light beams output from QWP2, which act as the signal arm of off-axis interference, are combined and off-axis interfered with a reference Gaussian beam by applying the BS3. The reference Gaussian beam propagates through a 3× beam expander composed by lens1 (f = 50 mm) and lens2 (f = 150 mm). A Pol. 2 restricts the reference beam to a 45°linearly polarized state. The expanded reference Gaussian beam then reflects at mirror3 and off-axis interferes with the signal beam. Then the off-axis interference light enters a loop composed by PBS3, mirror4, mirror5 and PBS4. This loop spatially separates the *x*-directional polarization component and *y*-directional polarization component of interference light fields, so the off-axis interference holograms of two polarization channels can be obtained by a charge-coupled device (CCD) in a single-shot detection. The captured off-axis interference holograms are then sent to a personal computer (PC) for digital processing to characterize the non-separability of generated vectorial structured light beam. In the experimental setup, QWP2 together with PBS3 splits the LCP channel and RCP channel, which corresponds to the polarization splitting process in Fig. 1a. It is worth mentioning that the two loops in this experimental setup can be simplified by Wollaston prisms or calcite beam displacers (e.g., Thorlabs, BD40).

### Non-separability characterization of vectorial structured light with *l* = 2

**Fig. 3:**
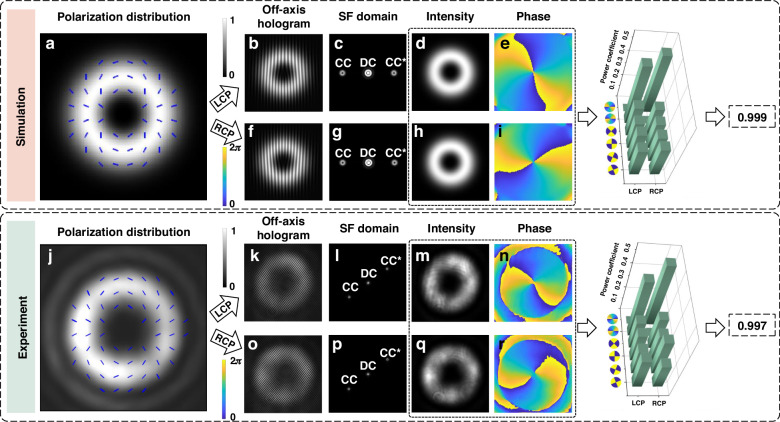
Simulation results and experimental results for characterizing the non-separability of the pure vector beam with a mode index of 2. **a** Simulated polarization distribution of the vector beam. **b–e** Simulated off-axis hologram, SF domain, retrieved intensity profile and phasefront of the LCP channel beam, respectively. **f–i** Simulated off-axis hologram, SF domain, retrieved intensity profile and phasefront of the RCP channel beam, respectively. **j** Experimentally measured polarization distribution of the vector beam. **k–n** Experimentally measured off-axis hologram, SF domain, retrieved intensity profile and phasefront of the LCP channel beam, respectively. **o–r** Experimentally measured off-axis hologram, SF domain, retrieved intensity profile and phasefront of the RCP channel beam, respectively. SF spatial frequency

Without loss of any generality, characterization of the non-separability of a pure vector beam with a mode index of 2 is accomplished, as indicated in Fig. 3. Figure 3a–i show all the simulation results to calculate the non-separability. The polarization distribution of the pure vector beam is simulated and given in Fig. 3a (see Supplementary Note 2 for more simulation details). The off-axis interference holograms of its LCP OV beam component and RCP OV beam component are simulated, as displayed in Fig. 3b, f. By performing 2-dimensional (2D) FFT on the off-axis holograms, SF domains in Fig. 3c, g are acquired. Filtering out CC terms and applying 2D IFFT on them enables fast retrieving the intensity profiles and phasefront of two OV beam components, as illustrated in Fig. 3d, e, h, i, respectively. With the known complex wavefront of two beam components in LCP and RCP channel, ${P}_{{i}L}$ and ${P}_{{i}R}$ can be obtained by calculating the overlapping degree between ideal wavefront of basic states and retrieved complex wavefront. Finally, a non-separability value of 0.999 is calculated by using Eqs. (2) and (3), which is very close to the set value 1.

Similarly, we experimentally demonstrate the non-separability characterization of the pure vector beam with a mode index of 2. Figure 3j indicates the experimentally retrieved polarization distribution of the pure vector beam (see Supplementary Note 3 for details). Figure 3k, o show the off-axis holograms captured by CCD. Correspondingly, their SF domains are displayed in Fig. 3l, p. Due to the experimental off-axis angle is obviously different from the simulation one, the distributions of experimental SF domains are quite distinguished from simulated SF domains. Experimentally retrieved intensity profiles and phasefront are illustrated in Fig. 3m, n, q, r, with the calculated ${P}_{{i}L}$ and ${P}_{{i}R}$ located at their right side. Experimentally, a non-separability value of 0.997 is characterized.

It seems that the interferograms in Fig. 3k, o feature lower contrast compared to reconstructed intensity profiles in Fig. 3m, q. This may due to the direct current term of the interference. That is, for a signal beam off-axis interfering with a reference beam, the intensity ${\left|{E}_{R}\left(x,y\right)\right|}^{2}$ of the reference beam always exists and acts as a background of the interferogram (see Materials and Methods), which leads to a decrease in contrast. For the reconstructed intensity profiles, this direct current term is removed, resulting in their high contrast.

It is worth noting that the successful operation of off-axis digital holography highly relies on reasonably choosing the tilt angle of light beams and the spatial resolution of detectors. According to the Nyquist–Shannon sampling theorem, the cut off frequency of the spatial-frequency is half the sampling frequency. Thus, if the spatial resolution of utilized detector is ∆*d*, the coinciding cutoff angular frequency is calculated as ${\omega }_{c}=\frac{2\pi }{2\Delta d}=\frac{\pi }{\Delta d}$ ^46^. This puts limitations on the tilt angle of off-axis interference: the spatial-frequency shift caused by the tilt angle should ensure that the CC term does not overflow the spatial-frequency domain (see Supplementary Note 9 for more details). In addition, the spatial-frequency shift caused by the tilt angle should enable separation between the CC term and the DC term.

**Fig. 4:**
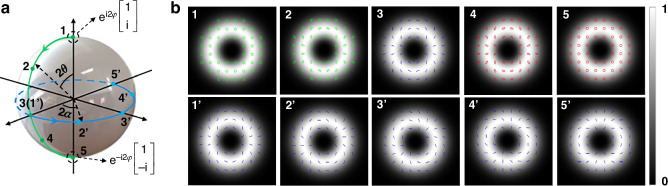
Different vectorial structured light beams on the HOPS for *l* = 2. **a** Geometric representation of vectorial structured light beams on the HOPS for *l* = 2. **b** Simulated polarization distributions of different vectorial structured light beams on the HOPS, the upper row corresponds to points 1-5 along the green solid line on the HOPS while the lower row corresponds to points 1’-5’ along the blue solid line on the HOPS. HOPS: higher-order Poincaré sphere

Next, we investigate the non-separability measurements of different vectorial structured light beams with *l* = 2 based on the proposed non-separability coherent detector. These vectorial structured light beams are, for a simple representation, different points on a higher-order Poincaré sphere (HOPS) for *l* = 2. Points on the HOPS for *l* = 2 can be thought as a superposition of two OV beams on the north pole and the south pole. For a geometric representation of these different vectorial structured light beams, they are featured by $(2\theta ,2\alpha )$ items of the spherical coordinate system, where $\theta$ decides the non-separability of the vectorial structured light beam while $2\alpha$ corresponds to the inter-modal angle in Eq. (1), as shown in Fig. 4a. Here, we vary $\theta$ and $2\alpha$ to investigate different vectorial structured light beams. Points along the green solid line connecting the north pole and the south pole represent vectorial structured light beams that possess different non-separability, obtained by varying $\theta \in [0,\pi /2]$ and maintaining $2\alpha =0$. Especially, the upper row of Fig. 4b indicates the simulated intensity profiles and polarization distributions of points 1-5 in Fig. 4a. In these figures which describe the polarization distributions of different vectorial structured light beams, green symbols represent LCP states, blue solid lines represent linearly polarized states, and red symbols represent RCP states, respectively. In addition, states on the equator of HOPS are investigated. These vectorial states are points 1’-5’ along the blue solid line in Fig. 4a, which can be acquired by varying $2\alpha \in [0,\pi ]$ while maintaining $\theta =\pi /4$. Intensity profiles and polarization distributions of vectorial structured light beams represented by points 1’-5’ are also simulated, as illustrated in the lower row of Fig. 4b. In the experiment, $\theta$ is controlled by changing the angle of Pol.1 (45° for pure vector beams and 0° for scalar OV beams, see Supplementary Note 4 for details) while $2\alpha$ is controlled by adjusting the phase difference of two phase holograms loaded onto the SLM.

**Fig. 5:**
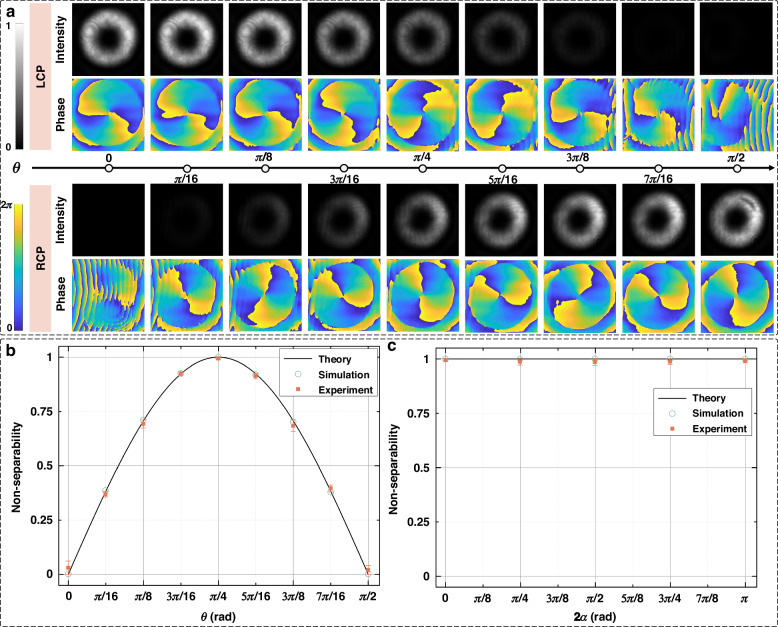
Results for characterizing the non-separability of different vectorial structured light beams. **a** Experimental results for characterizing the non-separability of vectorial structured light beams with different $\theta$. **b** Theoretically set non-separability, simulation detected non-separability and experimentally characterized non-separability for vectorial structured light beams with different $\theta$. **c** Theoretically set non-separability, simulation detected non-separability and experimentally characterized non-separability for vectorial structured light beams with different 2$\alpha$. For each case, we calculate the standard deviation of 50 measured non-separability results as the error bar

Figure 5 displays the results for characterizing the non-separability of different vectorial structured light beams with *l* = 2. Varying $\theta$ from 0 to $\pi /2$ while maintaining $2\alpha =0$, vectorial structured light beams with different non-separability $|\sin (2\theta )|$ can be obtained. In order to characterize the non-separability of these vectorial structured light beams, the off-axis interference holograms of their LCP components and RCP components are recorded. With the assistance of off-axis digital holography, the intensity profiles and phasefront of LCP and RCP components can be retrieved through the captured off-axis holograms, as illustrated in Fig. 5a. In Fig. 5a, the top two rows indicate the retrieved intensity profiles and phasefront of LCP components while the bottom two rows indicate the retrieved intensity profiles and phasefront of RCP components, with $\theta$ obeying a form of $n\pi /16$ which alters from 0 to $\pi /2$. Intensities of LCP components gradually decrease while intensities of RCP components gradually improve, when $\theta$ incrementally increases from 0 to $\pi /2$. Such a phenomenon can be explained by a glance at the power coefficients of ${{\bf{e}}}_{{\bf{L}}}$ and ${{\bf{e}}}_{{\bf{R}}}$ components in Eq. (1). Phasefront of LCP and RCP components possesses different vortex directions, showcasing that the LCP and RCP components are OV beams with opposite topological charges. It can be found that the retrieved phasefront deteriorates and even becomes wrong in some cases. It is because the signal-to-noise ratio (SNR) of captured off-axis holograms drops lower than the required threshold of off-axis digital holography in case that the intensities of off-axis holograms are too low.

Based on the retrieved intensity profiles and phasefront in Fig. 5a, non-separability of vectorial structured light beams which are defined by different $\theta$ are calculated, which can be found in Fig. 5b. In Fig. 5b, the black solid line represents the relationship of theoretical non-separability values versus $\theta$, while hollow circles and squares correspond to simulated non-separability and experimentally measured non-separability, respectively. For each experimental measurement, CCD quickly records 50 interferograms, which are sent to the digital calculation process. After digital calculation, we calculate the standard deviation of 50 non-separability results as the error bar of measurement. Meanwhile, we also experimentally achieve the non-separability characterization of vectorial structured light beams formed by varying the inter-modal angle of the superposition of LCP and RCP OV components. Figure 5c displays the relationships between the measured non-separability and $2\alpha$, in case that $2\alpha$ incrementally improves from 0 to $\pi$ and $\theta$ keeps a constant value of $\pi /4$. For the displayed experimental results in Fig. 5b, c (changing θ and 2α), the largest standard deviation of 50-time non-separability measurements is 0.0305, which demonstrates the high robustness of our proposed technique.

### Non-separability characterization of vectorial structured light superposition states

Since the demodulation part in conventional direct detection methods is replaced by calculating the overlapping degree between tested light beams and basic states in the proposed non-separability coherent detector, determining the non-separability of vectorial structured light superposition states is also efficient by means of adding more basic states in digital processing. Here, vectorial structured light superposition states which obey the following formula are investigated:

5 $${E}_{\mathrm{Sup}}=[\cos \,\gamma {E}_{OV}({l}_{1})+\,\sin \,\phi {E}_{OV}(-{l}_{2})]{{\bf{e}}}_{{\bf{L}}}+[\sin \,\gamma {E}_{OV}(-{l}_{1})+\,\cos \,\phi {E}_{OV}({l}_{2})]{{\bf{e}}}_{{\bf{R}}}$$

where ${E}_{Sup}$ represents the light field of a vectorial structured light superposition state. Such a light field is superimposed by two vectorial structured light beams with mode indices of ${l}_{1}$ and ${l}_{2}$, respectively. The vectorial structured light beam with a mode index of ${l}_{1}$ possesses non-separability of $|\sin (2\gamma )|$, while the other one contains non-separability of $|\sin (2\phi )|$. In the experiment, $\gamma$ and $\phi$ are controlled by fixing the angle of Pol.1 to 45°and adjusting the power ratio of ${l}_{1}$ OV component and ${l}_{2}$ OV component in each phase hologram loaded onto SLM (see Supplementary Note 5 for more details).

**Fig. 6:**
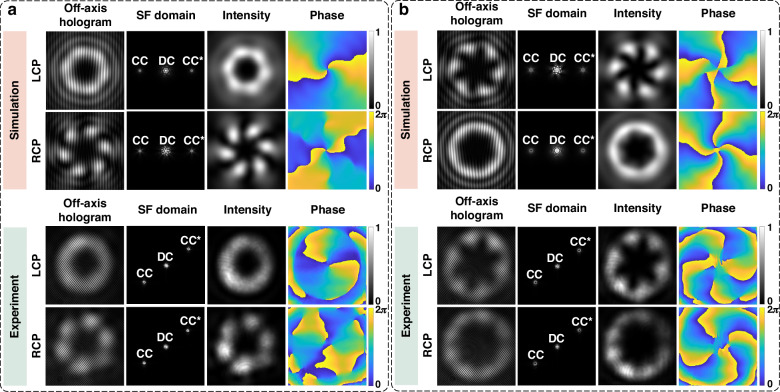
Simulation and experimental results for characterizing the non-separability of different vectorial structured light superposition states. **a** Simulation and experimental results for characterizing the non-separability of the superposition state with $\gamma =\pi /16,\phi =3\pi /16$. **b** Simulation and experimental results for characterizing the non-separability of the superposition state with $\gamma =\pi /5,\phi =\pi /16$

To start with, the non-separability characterizations of two-mode-superposition vectorial states are simulated and experimentally accomplished, as displayed in Fig. 6. For these two kinds of vectorial structured light superposition states, ${l}_{1}$ is set to 4 and ${l}_{2}$ equals 2. Figure 6a illustrates the concrete process and results for measuring the non-separability of a superposition state with $\gamma =\pi /16,\phi =3\pi /16$. The top two rows show the simulated off-axis holograms, SF domains, intensity profiles and phasefront of both the LCP component and RCP component to determine the non-separability of this superposition state. Correspondingly, the bottom two rows illustrate all the experimental results to characterize the non-separability of this superposition state. Considering another superposition state described by $\gamma =\pi /5,\phi =\pi /16$, simulation results and experimental results for characterizing its non-separability are indicated in Fig. 6b.

**Fig. 7:**
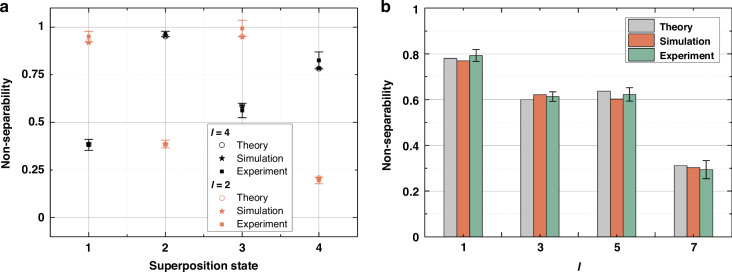
Results for characterizing the non-separability of different kinds of superposition states. **a** Characterizing the non-separability of four kinds of two-mode-superposition states. **b** Characterizing the non-separability of a four-mode-superposition states. The standard deviation of 50 measurements is plotted as an error bar

Except for the two superposition states in Fig. 6, non-separability characterization of another two vectorial structured light superposition states is also achieved. Non-separability detection results of these four superposition states are merged and displayed in Fig. 7a. Hollow circles represent theory values, solid stars are simulated non-separability, and solid squares correspond to experimentally characterized non-separability, respectively. Among them, superposition state 1 and superposition state 2 correspond to states in Fig. 6a, b, respectively. Superposition state 3 is defined by $\gamma =\pi /10,\phi =\pi /5$, while superposition state 4 is described by $\gamma =\pi /7,\phi =\pi /30$. For experimental results, we also record 50 off-axis holograms to calculate the standard deviation of measurement, which are shown as error bars in Fig. 7a.

Compared to characterizing the non-separability of a single mode, measuring the non-separability contributions of multiplex modes is more challenging. That is, for the case of multiple modes superposition, it is more difficult to accurately analyze the power weight of each mode through digital processing, which leads to errors in the analysis. Thus, when realistically characterizing such complex situations, it is necessary to ensure that the error is within an acceptable range. Through actual evaluations, we find that when using our method to measure the superposition of four modes, the error is relatively large but within an acceptable range. Figure 7b showcases results for measuring the non-separability of a four-mode-superposition state (see Supplementary Note 8 for details). One can see that although the error margins of such a superposition state is higher than results of a single vectorial mode, it is still within an acceptable range.

It is worth mentioning that, here the characterized non-separability of superposed vectorial state represents the non-separability contributions of modes with different $\left|l\right|$, which differs from the traditional space-polarization non-separability^30,34^. Only when the vectorial state is composed of OV modes in the same mode group, the non-separability contribution of this mode group is equivalent to the traditional space-polarization non-separability. For scenarios where such superposed vectorial states propagate through media that induce crosstalk between mode groups, the non-separability contributions cannot keep invariance, yet the space-polarization non-separability can (see Supplementary Note 10 for details). Thus, here we list some application scenarios for the characterized non-separability contributions. For instance, for media that only induce geometric phase (e.g., out-plane perturbations of optical fibers), the non-separability contributions of different modal groups can keep invariance. Moreover, such non-separability contributions can be applied to quantitively characterize the spin-orbit coupling within specific modal groups. For optical communication or optical sensing systems based on multi-mode fibers (MMFs), intra-modal-group crosstalk is much more likely to occur than inter-modal-group crosstalk, since the effective refractive index difference within the mode group is much smaller. In this case, our technique may be useful to provide more information within specific modal groups. For example, a measured non-separability contribution of 1 represents the worst situation, i.e., crosstalk of the input pure mode within the mode group is completely random.

## Discussion

In summary, a coherent detector for the non-separability measurement of vectorial structured light is proposed and experimentally demonstrated. The coherent detector splits two circularly polarized components of vectorial structured light and reconstructs their complex phasefront based on off-axis digital holography. Calculating the overlapping degree between ideal complex phasefront of different basic states and the retrieved complex phasefront enables characterizing the intensity coefficients of different basic states to obtain the non-separability. Different from traditional non-separability detection methods that directly measure the light intensity, the coherent detector proposed in this paper detects the phasefront of light beams to characterize the non-separability. Due to the completely different principle, the proposed coherent detector features lots of advantages compared to traditional direct detection methods.

Here, a comparison between the efficiency of traditional direct detection methods and the proposed coherent detector is investigated, as indicated in Table 1. The performance of these methods is mainly investigated in four aspects. That is, are they single-shot? How about the spatial complexity of the detection setup (we define the requested number of spatial detection channels on the detector as the spatial complexity)? Does the detection setup require SLM/DMD? Are they capable of detecting vectorial structured light superposition states? Traditional direct detection method for the non-separability contains basis-dependent approaches and basis-independent approaches. As illustrated in Table 1, basis-dependent approaches can be divided into two kinds: switch demodulating holograms to demodulate tested beams over different basic modes^29,34,37,38^, or use a demultiplexing hologram to demodulate different modes to different locations at the same time^37,39^. For the former basis-dependent methods, although they possess the advantage of low spatial complexity, switching demodulate holograms means that SLM/DMD is required and these methods are not single-shot. For the latter basis-dependent methods, they feature a superiority of single-shot characterization, but they are limited by their high spatial complexity and requirement for SLM/DMD. The basis-independent methods measure the Stokes parameters of vectorial structured light field to characterize its non-separability^34,36^. These methods own advantages of single-shot and SLM/DMD-free detection, yet it also keeps a relatively high spatial complexity of 4. In addition, non-separability characterization of vectorial structured light superposition states based on all these traditional direct detection methods has not been accomplished. In contrast, the non-separability coherent detector proposed in this paper possesses the advantages of single-shot and SLM/DMD-free detection, a relatively low spatial complexity of 2, and the ability to detect the non-separability of structured light superposition states. Due to the superiorities of proposed non-separability coherent detector, it may pave the way for turbulence-resilient optical communications based on non-separability coding methods^35,36^ and provide an efficient tool for classical entanglement based on non-separability of light^53–55^. For instance, many complex non-separable states with higher-dimensional classical entanglement have been proposed and experimentally created^42–47^. Since our technique enables efficiently detecting the non-separability of vectorial light fields with only two interferograms, it may be capable of dealing with the non-separability measurement of such complex non-separable states.

**Table 1:** Comparison between traditional methods and the proposed coherent detector

Reference	Principle	Single-shot?	Spatial complexity	Require SLM/DMD?	Superposition state detection
^29,34,37,38^	Basis-dependent: Switch demodulating holograms	No	1	Yes	No
^37,39^	Basis-dependent: Demultiplexing hologram	Yes	12 for ref. ^37^ 8 for ref. ^39^	Yes	No
^34,36^	Basis-independent: Stokes parameters measurement	Yes	4	No	No
This work	Coherent detection: Off-axis holography	Yes	2	No	Yes

Remarkably, there are already some works that characterize vectorial structured light beams^56,57^, even also by reconstructing the amplitude distributions and phasefronts of vectorial structured light^56^. However, our work is different from them. That is, such work detects the Stokes parameters of vectorial structured light beams to retrieve their polarization distributions^57^. Moreover, polarization-sensitive cameras are also able to efficiently detect the polarization distributions of light fields. However, although the polarization distributions/full Stokes parameters of light fields are given, one cannot perform the digital modal decomposition process to characterize the non-separability of vectorial structured light beams (see Supplementary Note 11). Our technique enables not only achieving measuring the polarization distribution of a vectorial structured light beam but also retrieving its modal information in orthogonal polarization channels.

Moreover, it seems that mode sorters are also able to efficiently decompose different spatial modes. However, generally speaking, a mode sorter (e.g., coordinate transformation schemes, multi-plane light conversion devices, Dammann gratings, mode-selective couplers, etc) is usually designed to sort orthogonal spatial modes. For the modal tomography process in non-separability characterization, high-efficient sorting of non-orthogonal spatial modes is required. For a single mode sorter, the modal tomography process means simultaneously sorting two orthogonal modes ${E}_{OV}(l)$, ${E}_{OV}(-l)$, and their non-orthogonal modes ${E}_{OV}(l)+{e}^{i\beta }{E}_{OV}(-l)$, which may induce high modal crosstalk. Even if a mode sorter can achieve sorting of these non-orthogonal modes with tolerable mode crosstalk, it can only be efficient within a limited number of mode groups. For instance, if a mode sorter can efficiently sort the six modes within the 1^st^ mode group, it is difficult to maintain effectiveness when the sorting target is changed to 10^th^ mode group. Yet for our scheme, the sorting process is realized through a digital way, ensuring that it is efficient for any mode group. Thus, compared to the approach using a mode sorter, our technique intrinsically possesses higher efficiency and scalability.

Although our technique possesses several advantages, there are also a limitation on it. That is, our technique places requirements on the coherence of utilized laser source. In our experiment, a coherent laser with a coherence length of about 100 m is utilized. A high coherence performance of the laser source ensures a high interference contrast during non-separability measurements. In addition, for practical long-haul atmospheric turbulence systems, acquisition of the reference beam may be difficult, since the laser is in situ and the vectorial structured light is transmitted to the remote end. Such a problem may be solved by common-path self-reference holography techniques. For instance, one may use a single-mode fiber which acts as a spatial filter to receive part of the vectorial structured light beams to create a reference Gaussian beam (see Supplementary Note 7 for more details). Moreover, it is worth to mention that our technique is targeted for applications in atmospheric turbulence. For strongly scattering media which induce polarization-dependent delays, it may decoherent the transmitted light beams, leading to a failure of off-axis digital holography.

## Materials and Methods

### Off-axis digital holography

Off-axis digital holography enables capturing the complex wavefront of beam in a single-exposure acquisition. For a signal beam, its light field can be expressed as $\left|{E}_{S}\left(x,y\right)\right|{e}^{i{\varphi }_{S}\left(x,y\right)}$, where $\left|{E}_{S}\left(x,y\right)\right|$ is the amplitude distribution and ${\varphi }_{S}\left(x,y\right)$ is its phasefront. For an off-axis reference beam, its light field can be written as ${E}_{R}(x,y)=\left|{E}_{R}(x,y)\right|{e}^{i[{\varphi }_{R}(x,y)-2\pi {u}_{0}x]}$, where $\left|{E}_{R}(x,y)\right|$ denotes its amplitude distribution, ${\varphi }_{R}(x,y)$ represents its phasefront and $-2\pi {u}_{0}x$ corresponds to *x*-directional tilt. Thus, the intensity of off-axis interferogram can be written as,

6 $$\begin{array}{l}{\left|{E}_{inter}\left(x,y\right)\right|}^{2}=\left[{E}_{S}\left(x,y\right)+{E}_{R}\left(x,y\right)\right]\cdot {\left[{E}_{S}\left(x,y\right)+{E}_{R}\left(x,y\right)\right]}^{* }\\ ={\left|{E}_{S}\left(x,y\right)\right|}^{2}+{\left|{E}_{R}\left(x,y\right)\right|}^{2}+\left|{E}_{S}\left(x,y\right)\right|\left|{E}_{R}\left(x,y\right)\right|{e}^{i\left[{\varphi }_{S}\left(x,y\right)-{\varphi }_{R}\left(x,y\right)+2{\rm{\pi }}{u}_{0}x\right]}+\left|{E}_{S}\left(x,y\right)\right|\left|{E}_{R}\left(x,y\right)\right|{e}^{i\left[-{\varphi }_{S}\left(x,y\right)+{\varphi }_{R}\left(x,y\right)-2{\rm{\pi }}{u}_{0}x\right]}\end{array}$$

where ${\left|{E}_{S}\left(x,y\right)\right|}^{2}+{\left|{E}_{R}\left(x,y\right)\right|}^{2}$ is the DC term of the off-axis interference, and the last two terms correspond to CC and CC^*^. The phase term 2π*u*_0_*x* and -2π*u*_0_*x* within this equation enable CC and CC^*^ shifting in spatial-frequency domain. Applying a 2D FFT to the interference holograms and seeing in the SF domain, the three DC, CC and CC^*^ terms are completely separated,

7 $${\mathcal{F}}\left\{{\left|{E}_{{inter}}\left(x,y\right)\right|}^{2}\right\}={DC}\left(u,y\right)+{CC}\left(u-{u}_{0},y\right)+{{CC}}^{* }\left(u+{u}_{0},y\right)$$

where$\,\text{DC}\left(u,y\right)$, *CC*$\left(u-{u}_{0},y\right)$ and CC^*^$\left(u+{u}_{0},y\right)$ represent the Fourier transform of the DC, CC and CC^*^ terms. After filtering out the CC term, moving it to the center of spatial-frequency domain and performing inverse FFT on it, we can finally obtain the reconstructed amplitude distribution $\left|{E}_{S}\left(x,y\right)\right|\left|{E}_{R}\left(x,y\right)\right|$ and phasefront ${\varphi }_{S}\left(x,y\right)-{\varphi }_{R}\left(x,y\right)$,

8 $${{\mathcal{F}}}^{-1}\left\{{CC}\left(u,y\right)\right\}=\left|{E}_{S}\left(x,y\right)\right|\left|{E}_{R}\left(x,y\right)\right|{e}^{i\left[{\varphi }_{S}\left(x,y\right)-{\varphi }_{R}\left(x,y\right)\right]}$$

In the experiment, we expand a Gaussian beam into a nearly plane wave as the reference beam, that is, $\left|{E}_{R}\left(x,y\right)\right|$ and ${\varphi }_{R}\left(x,y\right)$ can be thought as constants.

## Supplementary information

Supplementary Information

## Data Availability

All data, theory details, simulation details that support the findings of this study are available from the corresponding authors upon reasonable request.
