# Supplementary material for: Coherent detector for the non-separability measurement of vectorial structured light
Source: Light Sci Appl. 2025 Sep 26;14:343. doi: 10.1038/s41377-025-02035-1 (PMC12475189; doi:10.1038/s41377-025-02035-1)
Supplement: Supplementary file 1 — Supplementary Information [file 41377_2025_2035_MOESM1_ESM.pdf]

Supplementary Information: Coherent detector for the non-separability measurement of vectorial structured light

Yize Liang[1,2,3], Shuai Cao[1], Lixian Liu[1], Fei Liu[1], Xukun Yin[1], Pei Lv[1], Yiming Zhang[1], Yunrui Zou[1], Liang Fang[4], Shuang Zheng[2,3], Zhenyu Wan[2,3], Teli Xi[1,*], Xiaopeng Shao[5,*], and Jian Wang[2,3,6*]

[1] School of Optoelectronic Engineering, Xidian University, Xi'an 710071, China

[2] Wuhan National Laboratory for Optoelectronics and School of Optical and Electronic Information, Huazhong University of Science and Technology, Wuhan 430074, Hubei, China

[3] Optics Valley Laboratory, Wuhan 430074, Hubei, China

[4] School of Physics and Electronics, Hunan University, Changsha 410082, China

[5] Xi'an Institute of Optics and Precision Mechanics, CAS, Xi'an 710119, China

[6] Hubei Optical Fundamental Research Center,Wuhan 430074, China

* Correspondence to: jwang@hust.edu.cn, tlxi@xidian.edu.cn, xpshao@opt.ac.cn

Supplementary Note 1. Introduction

In this supplementary information, we investigate more details and results to further explain our non-separability coherent detector. Except for the results shown in the Article, we show: (I) principle and details for the simulation results in the Article; (II) process and experimental results for reconstructing the polarization distribution of the vectorial structured light beam in Fig. 3j in the Article; (III) method for experimentally controlling the θ and 2α of vectorial structured light beams generated by the experimental setup shown in Fig. 2 in the Article; (IV) approach to experimentally controlling the non-separability of vectorial structured light superposition states; (V) additional results for simulating and experimentally characterizing the non-separability of vectorial structured light superposition states 3 and 4 in Fig. 7 in the Article; (VI) Common-path self-reference scheme for the proposed off-axis digital holography technique; (VII) Additional results for characterizing a four-mode-superposition vectorial state; (VIII) Operation condition and limitations of off-axis digital holography; (IX) Simulation results for propagating a superposition vectorial state through atmospheric turbulence; (X) Theoretical proof and simulation results which demonstrate that Stokes parameters can't be utilized to accomplish the digital modal decomposition process.

These ten parts are organized as Supplementary Note 2 to Supplementary Note 11 respectively.

Supplementary Note 2. Simulation principle and details

In the Article, polarization distributions and non-separability characterizations of vector beams are simulated. As indicated in Fig. S1, here we claim the detailed process for achieving such a simulation.

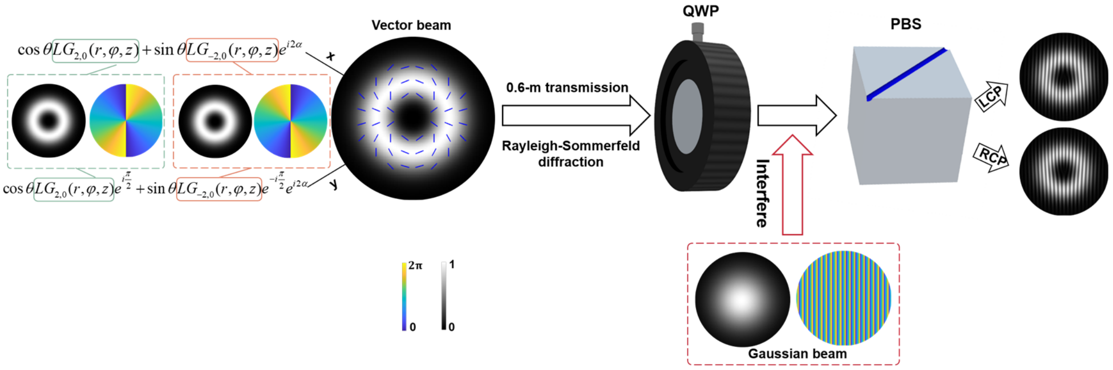

Fig. S1. Detailed process for the simulation to determine the non-separability of vector beams.

Without loss of any generality, vectorial structured light beam which possesses a mode index of l=2 is taken as an example. Such a vectorial structured light beam is composed by two optical vortex beams with opposite spin and orbital angular momenta. Here, we utilize Laguerre-Gaussian (LG) beams with

radial indices p=0 to represent the two optical vortex beams. The scalar electric field of a $LG_{l,p}$ beam in cylindrical coordinates can be described by[1,2]

$$LG_{l,p}(r,\varphi,z)=\left(\frac{2p!}{\pi\left(p+|l|\right)!}\right)^{1/2}\frac{1}{w(z)}\left[\frac{r\sqrt{2}}{w(z)}\right]^{|l|}\exp\left[-\frac{r^2}{w^2(z)}\right]L_p^{|l|}\left(\frac{2r^2}{w^2(z)}\right)$$
$$\exp\left[-i\frac{k_0r^2z}{2\left(z^2+z_R^2\right)}\right]\exp\left[-i\left(2p+|l|+1\right)\arctan\left(\frac{z}{z_R}\right)\right]\exp\left(il\varphi\right) \tag{s1}$$

where (r,φ,z) are the cylindrical coordinates, $w(z)$ defines the $1/e$ radius at distance z and $w(z)=w_0\left[\left(z^2+z_R^2\right)/z_R^2\right]^{1/2}$, z_R is the Rayleigh range, $k_0=\frac{2\pi}{\lambda}$ is the wave number of light in vacuum, $L_p^{|l|}(x)$ is an associated Laguerre polynomial, obtained from the more familiar Laguerre polynomials by:

$$L_p^{|l|}(x)=(-1)^{|l|}\frac{d^{|l|}}{dx^{|l|}}L_{p+|l|}(x) \tag{s2}$$

As one of the two components of vector beams described by Eq. () in the Article, a left circularly polarized (LCP) $LG_{2,0}$ beam can be described as:

$$\begin{cases}E_x=\cos\theta LG_{2,0}(r,\varphi,z)\\ E_y=\cos\theta LG_{2,0}(r,\varphi,z)e^{i\frac{\pi}{2}}\end{cases} \tag{s3}$$

Similarly, a right circularly polarized (RCP) $LG_{-2,0}$ beam can be described as:

$$\begin{cases}E_x=\sin\theta LG_{-2,0}(r,\varphi,z)\\ E_y=\sin\theta LG_{-2,0}(r,\varphi,z)e^{-i\frac{\pi}{2}}\end{cases} \tag{s4}$$

In the simulation, in order to efficiently determine the Jones vectors of pixels of light fields to plot their polarization distributions, all the light fields are emulated in x and y polarization channels. Assuming that the LCP $LG_{2,0}$ beam and RCP $LG_{-2,0}$ beam are superposed with a phase difference 2α , the synthesized vector beam can be written as:

$$\begin{cases}E_{v,x}=\cos\theta LG_{2,0}(r,\varphi,z)+\sin\theta LG_{-2,0}(r,\varphi,z)e^{i2\alpha}\\ E_{v,y}=\cos\theta LG_{2,0}(r,\varphi,z)e^{i\frac{\pi}{2}}+\sin\theta LG_{-2,0}(r,\varphi,z)e^{-i\frac{\pi}{2}}e^{i2\alpha}\end{cases} \tag{s5}$$

Then we let the two polarization components of such a vector beam transmits 0.6 m to better emulate the beam transmission effect in the experiment. For the beam transmission simulation, we choose Rayleigh-Sommerfeld diffraction numerical integration. After 0.6-m transmission, the x and y components of the vector beams change to $U_{v,x}$ and $U_{v,y}$, respectively.

To characterize the non-separability of the vector beam, polarization sorting of two circularly polarized components is required. In the experiment, the polarization sorting is accomplished by using a combination of a 45° quarter-wave plate (QWP) and a polarization beam splitter (PBS). Applying the Jones matrix of 45° QWP, light beam after propagating through the QWP can be expressed as:

$$\begin{bmatrix} 1 & i \\ i & 1 \end{bmatrix} \begin{bmatrix} U_{v,x} \\ U_{v,y} \end{bmatrix} = \begin{bmatrix} U_{v,x} + iU_{v,y} \\ iU_{v,x} + U_{v,y} \end{bmatrix} \tag{s6}$$

PBS separates the x polarization component and y polarization component of light beams. Thus, in our simulation, the two beams split by PBS can be described as: $\begin{bmatrix} U_{v,x} + iU_{v,y} \\ 0 \end{bmatrix}$ and $\begin{bmatrix} 0 \\ iU_{v,x} + U_{v,y} \end{bmatrix}$. Then the off-axis interferences of these two beams are simulated by interfering each of them with an off-axis Gaussian beam. The off-axis Gaussian beam is simulated as $E_{Gaussian} e^{i\frac{2\pi x}{d}}$, where $E_{Gaussian}$ is the electric field of on-axis Gaussian beam, $e^{i\frac{2\pi x}{d}}$ is an x-directional grating which leads to x-directional diffraction angle, d is the period of such a grating. After acquiring the off-axis holograms of two polarization channels, complex wavefront of light beams can be reconstructed, obeying the process displayed in Fig. 3 in the Article. Then one can calculate the overlapping degree between reconstructed light fields and six basic states to determine the non-separability of simulated vectorial structured light beams.

The above descriptions detail the process for simulating the light field of a pure vectorial structured light beam. When simulating vectorial structured light superposition states, considering SLM only offers phase modulation, we choose to use $E_{Gaussian} \cdot e^{il\varphi}$ instead of the scalar electric field of a pure $LG_{l,0}$ beam to emulate experimental scenarios more realistically.

In the simulation, the polarization distributions of beams are characterized by calculating the Jones vectors of each pixel of light field. Taking the vector beam with a mode index of l=2 as an instance, Jones vectors of each pixel of such a vector beam can be computed, since the electric field of x-polarized and

y-polarized components are already known in Eq. (s5). In our simulation, light fields possess a resolution of 300×300. In order to plot polarization distributions onto intensity profiles of light fields, we divide the light field into 30×30 superpixels. Each superpixel is a 10×10 matrix. The Jones vector value of each superpixel is obtained by averaging the value of Jones vectors of inside 10×10 pixels. For the vector beam shown in Fig. S1, Figure S2 (a) illustrates the calculated 30×30 matrix of Jones vectors. The upper row in Fig. S2 (a) shows the amplitude and phase of the x component of the Jones vectors, while the lower row indicates the amplitude and phase of the y component of the Jones vectors.

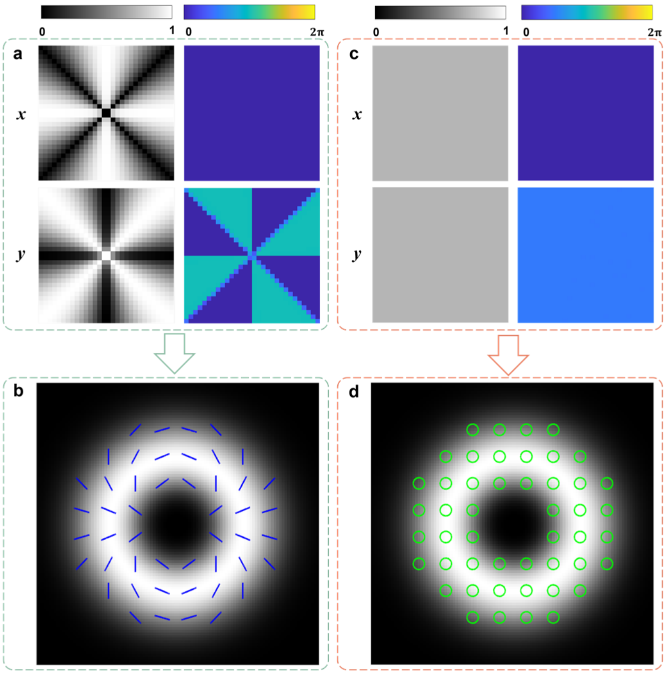

Fig. S2. Reconstructing the polarization distribution of light beams in simulation. a, x and y component of Jones vectors of the vector beam in Fig. S1. **b,** Reconstructed polarization distribution of the pure vector beam. **c,** x and y component of Jones vectors of an LCP $LG_{2,0}$ beam. **d,** Reconstructed polarization distribution of the LCP $LG_{2,0}$ beam.

Based on the calculated Jones vectors, the polarization of each superpixel can be described by the simple harmonic oscillation function in two orthogonal directions:

$$\begin{cases} E_x(i,j) = |Jones_x(i,j)|\cos(\phi) \\ E_y(i,j) = |Jones_y(i,j)|\cos(\phi + \arg(Jones_y(i,j))) \end{cases} \tag{s7}$$

where $Jones_x(i,j)$ and $Jones_y(i,j)$ are the x or y component of Jones vector of (i,j) superpixel respectively, ϕ defines the transmission and oscillation phase of light which is usually written as

$kz-\omega t+\varphi_0$. Taking the value of ϕ from 0 to 2π enables plotting the polarization of (i,j) superpixel. When plotting polarizations onto intensity profiles of light fields, only polarizations of superpixels with relatively high intensity are plotted. For instance, polarization distribution of the vector beam in Fig. S2b can be reconstructed based on calculated Jones vectors in Fig. S2a. Similarly, Fig. S2c indicates the *x* and *y* component of Jones vectors of an LCP $LG_{2,0}$ beam. The reconstructed polarization distribution is shown in Fig. S2d. According to the positive, zero or negative value of $\arg(Jones_y(i,j))$, one can judge whether the polarization of (i,j) superpixel is LCP, linearly-polarized (LP) or RCP. In the Article, green, blue and red symbols correspond to LCP, LP and RCP, respectively.

Supplementary Note 3. Experimentally retrieve the polarization distributions of vector beams

Polarization distribution of a pure vector beam is retrieved, as illustrated in Fig. 3j in the Article. Here we show the principle, concrete process and experimental results for retrieving the polarization distribution of this pure vector beam. The polarization distribution of a light beam can be described by its Stokes parameters. All the four Stokes parameters of a light beam can be calculated by utilizing the following formula[3,4],

$$\begin{cases} S_0(x,y)=I_X(x,y)+I_Y(x,y) \\ S_1(x,y)=I_X(x,y)-I_Y(x,y) \\ S_2(x,y)=I_{+45}(x,y)-I_{-45}(x,y) \\ S_3(x,y)=I_R(x,y)-I_L(x,y) \end{cases} \tag{s8}$$

where (x,y) represents the Cartesian coordinates of light beam transverse plane, $S_0(x,y)$ - $S_3(x,y)$ denote the distribution of four Stokes parameters, $I_X(x,y)$, $I_Y(x,y)$, $I_{+45}(x,y)$ and $I_{-45}(x,y)$ correspond to intensity profiles of light beam transmitting through a horizonal, vertical, +45° and -45° polarizer, respectively. $I_R(x,y)$ corresponds to intensity profiles of light beam propagating through a +45° QWP and a horizonal polarizer while $I_L(x,y)$ corresponds to intensity profiles of light beam passing through a +45° QWP and a vertical polarizer. $I_L(x,y)$ and $I_R(x,y)$ describe the value of LCP and RCP components of each point on the light beam transverse plane. Thus, four Stokes parameters of all the points of light beam are measured using such six intensity profiles. The measured

four Stokes parameters determine the polarization of each point of beam at the transverse plane. Illustrated in Fig. S3 are all the experimental results for retrieving the polarization distribution of the pure vector beam in Fig. 3j in the Article. Figure S3 shows the retrieved polarization distribution, while Figs. S3b-g display the intensity profiles of $I_X(x,y)$, $I_Y(x,y)$, $I_{+45}(x,y)$, $I_{-45}(x,y)$, $I_L(x,y)$ and $I_R(x,y)$.

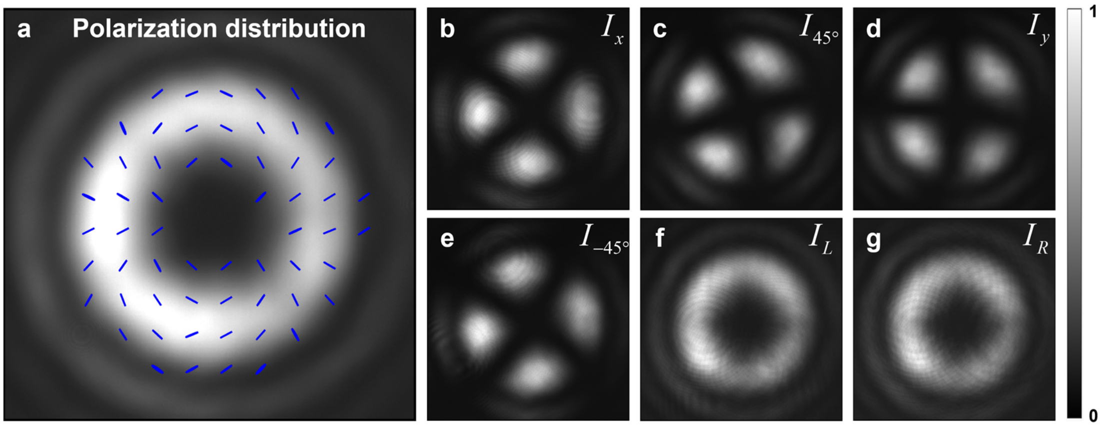

Fig. S3. Experimentally results for retrieving the polarization distribution of vectorial structured light beam in Fig. 3 (j) in the Article. a, Retrieved polarization distribution of the vectorial structured light beam. **b-g,** Recorded intensity profiles I_x, $I_{45°}$, I_y, $I_{-45°}$, I_L and I_R for retrieving the polarization distribution of the vectorial structured light beam, respectively.

After obtaining all the four Stokes parameters of points on the light beam transverse plane, the next goal is marking polarization distributions on the captured intensity profile of light. Here, the concept of superpixel is also used. For instance, the captured intensity profile of Fig. S3a possesses 960×960 pixels, so it is obviously impossible to mark the polarizations of each point on this intensity profile. Hence, the 960×960 intensity profile is separated to 12×12 superpixels with each superpixel containing 80×80 pixels, during our processing. Calculating the Stokes parameters of 12×12 superpixels enables retrieving their polarizations. Once the Stokes parameters of the 12×12 superpixels are measured, polarization of each superpixel can be marked based on the following equation[5],

$$\begin{cases} E_X = \sqrt{(1+S_1/S_0)/2} * \cos(\varphi) \\ E_Y = \sqrt{(1-S_3/S_2)/2} * \cos[\varphi + \tan^{-1}(S_3/S_2)] \end{cases} \tag{s9}$$

where E_X, E_Y describe the simple harmonic vibration functions of light in two orthogonal directions, they decide the trajectory of light beam vector. φ represents the transmission and vibration phase of light,

usually described as $\varphi = k \cdot z - \omega \cdot t + \varphi_0$, which takes the same value for two orthogonal directions. The trajectory of beam vector determined by E_X and E_Y can be calculated by taking the value of φ from 0 to 2π in this parameter equation system about φ. That is, the polarization of superpixel can be marked through varying φ from 0 to 2π and plotting E_X and E_Y. In our experimental processing, for a clearer display, only the polarization of superpixel whose intensity is higher than a given threshold is marked.

Supplementary Note 4. Control the θ and 2α of generated vectorial structured light beams

In the experiment, θ and 2α of generated pure vectorial structured light beams are controlled to investigate the efficiency of proposed non-separability coherent detector, as indicated in Fig. 5 in the Article. Since Pol. 1 with an angle of θ in Fig. 2 in the Article results in a Jones matrix of $\begin{bmatrix} \cos\theta \\ \sin\theta \end{bmatrix}$ of light beam passing through it, power ratio between LCP channel and RCP channel is controlled by adjusting the angle of Pol. 1. The finally composed vectorial structured light beam can be expressed by,

$$V(r,\theta) = \cos\theta e^{il\varphi} \begin{bmatrix} 1 \\ i \end{bmatrix} + \sin\theta e^{-il\varphi} \begin{bmatrix} 1 \\ -i \end{bmatrix} \qquad \text{(s10)}$$

which showcases that the non-separability of vectorial structured light beam is controlled by the angle θ of Pol. 1. The 2α term describes the inter-modal phase of two OV beam components which compose the vectorial structured light beam, as defined in Eq. (1) in the Article. In the experiment, 2α is controlled by altering the phase difference between two holograms loaded onto the SLM. Hence, two phase holograms which control the value of 2α can be described by,

$$\text{phase1} : \text{mod}(l\varphi + \text{grating}, 2\pi) \qquad \text{phase2} : \text{mod}(-l\varphi + \text{grating} + 2\alpha, 2\pi) \qquad \text{(s11)}$$

where phase1 and phase2 denote two phase holograms loaded onto the SLM, grating corresponds to a horizonal blazing grating phase $i2\pi x/d$ which separates the modulated beam and unmodulated beam for SLM, mod represents the remainder operation.

Supplementary Note 5. Experimentally control the non-separability of vectorial structured light superposition states

In this section, we show the principle and phase holograms utilized to control the non-separability of vectorial structured light superposition states. Considering a vectorial structured light superposition state defined by the following formula,

$$\begin{cases} E_{\text{Sup\_LCP}} = \cos\gamma E_{OV}(l_1) + \sin\phi E_{OV}(-l_2) \\ E_{\text{Sup\_RCP}} = \sin\gamma E_{OV}(-l_1) + \cos\phi E_{OV}(l_2) \end{cases} \tag{s12}$$

where $E_{\text{Sup\_LCP}}$ represents the light field of LCP component of the superposition state while $E_{\text{Sup\_RCP}}$ denotes the RCP one. This formula defines that: the vectorial structured light beam with a mode index of l_1 possesses non-separability of $|\sin(2\gamma)|$, while the other one contains non-separability of $|\sin(2\phi)|$. To generate the superposition state described by Eq. (s12), we investigate two phase holograms loaded onto the SLM:

$$\begin{aligned} &\text{phase1}: \text{mod}[(p_1 l_1\varphi - p_2 l_2\varphi) + \text{grating}, 2\pi] \\ &\text{phase2}: \text{mod}[(-\tan\gamma p_1 l_1\varphi + \cot\phi p_2 l_2\varphi) + \text{grating}, 2\pi] \end{aligned} \tag{s13}$$

where p_1 denotes the percentage of phase $l_1\varphi$ in phase1 while p_2 denotes the percentage of phase $-l_2\varphi$ in phase1. Noting that weights of different OV phase in the same phase hologram sum to 1, we have,

$$\begin{aligned} &p_1 + p_2 = 1 \\ &\tan\gamma p_1 + \cot\phi p_2 = 1 \end{aligned} \tag{s14}$$

From Eq. (s14) we can calculate the value of p_1 and p_2:

$$\begin{aligned} p_1 &= \frac{\cot\phi - 1}{\cot\phi - \tan\gamma} \\ p_2 &= \frac{1 - \tan\gamma}{\cot\phi - \tan\gamma} \end{aligned} \tag{s15}$$

Therefore, two phase holograms loaded onto the SLM are decided when γ and ϕ are given, as shown in Fig. S4. Figure S4a displays the concrete process for computing the phase hologram for the superposition state 1 in Fig. 7 in the Article. Firstly, a superposition of p_1 multiplies phase of OV_4 and p_2 multiplies phase of OV_{-2} constitutes the basic phase of phase1. Secondly, a superposition of $\tan\gamma p_1$ multiplies phase of OV_{-4} and $\cot\phi p_2$ multiplies phase of OV_2 forms the basic phase of phase2. Finally, grating phase is superimposed. Considering that a reflective SLM is applied in our experiment, a phase conjugation is performed on the computed phase. Similarly, Figs. S4b-d showcase the phase holograms for generating superposition state 2, 3 and 4 in Fig. 7 in the Article.

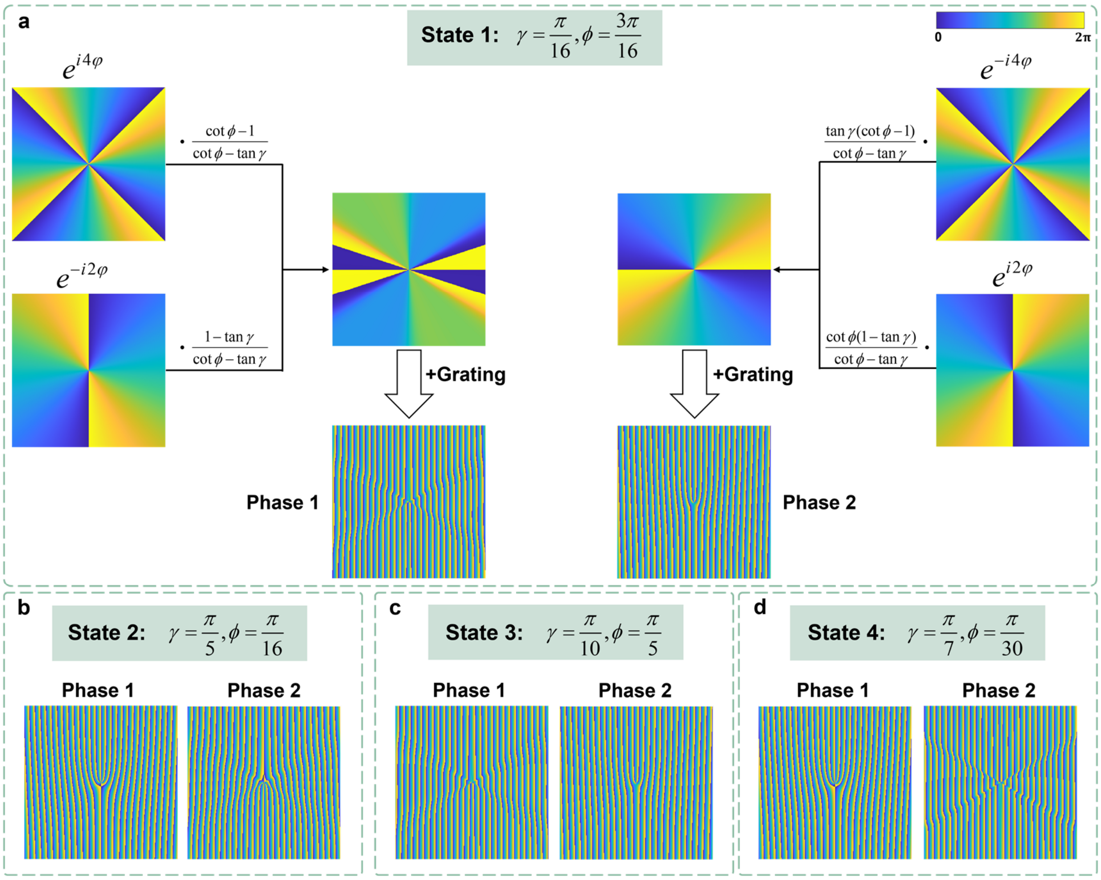

Fig. S4. Phase holograms for generating different vectorial structured light superposition states. a, Process to determine two phase holograms for the generation of vectorial structured light superposition state 1. **b-d,** Phase holograms for generating superposition states 2-4, respectively.

Supplementary Note 6. Additional results for simulating and experimentally characterizing the non-separability of vectorial structured light superposition states 3 and 4

In this part, we show additional results for simulating and experimentally characterizing the non-separability of vectorial structured light superposition states 3 and 4 in Fig. 7 in the Article. Fig. S5a illustrates the concrete process and results for measuring the non-separability of the superposition state 3 with $\gamma=\pi/10,\phi=\pi/5$. The top two rows show the simulated off-axis holograms, SF domains, intensity profiles and phasefront of both the LCP component and RCP component to determine the non-separability of the superposition state 3. Correspondingly, the bottom two rows illustrate all the experimental results to characterize the non-separability of the superposition state 3. As a result, the superposition state 3 possesses a theoretical non-separability value of 0.588 for l_1 and 0.951 for l_2, simulated non-separability value of 0.587 for l_1 and 0.950 for l_2, experimentally characterized non-

separability value of 0.615 for l_1 and 0.990 for l_2. For the superposition state 4 described by $\gamma = \pi/7, \phi = \pi/30$, simulation results and experimental results for characterizing its non-separability are indicated in Fig. S5b. In this case, the superposition state possesses a theoretical non-separability value of 0.782 for l_1 and 0.208 for l_2, simulated non-separability value of 0.785 for l_1 and 0.172 for l_2, experimentally characterized non-separability value of 0.808 for l_1 and 0.161 for l_2.

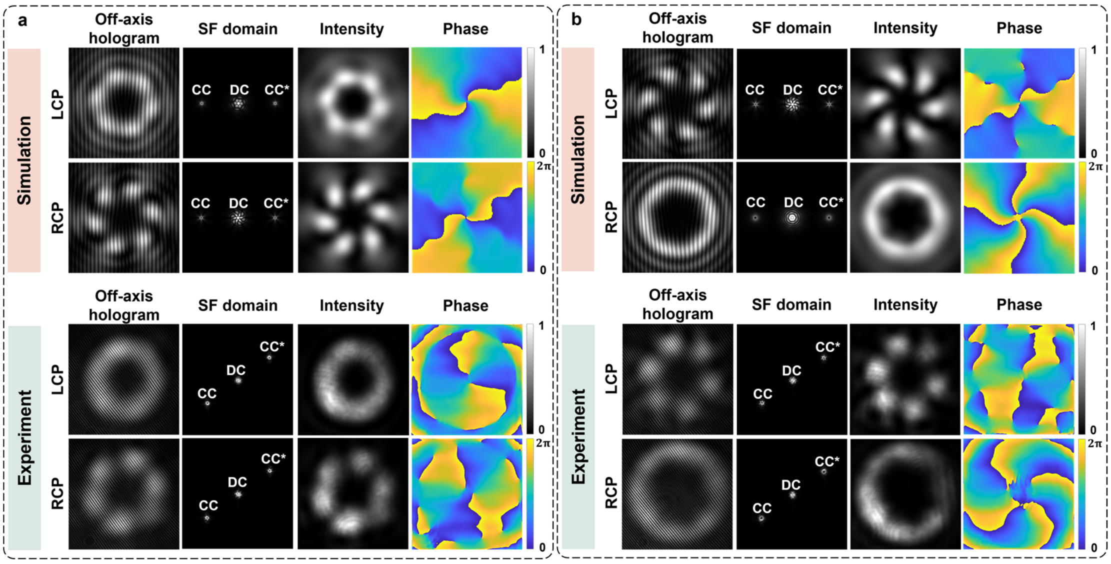

Fig. S5. Additional results for characterizing the non-separability of superposition state 3 and 4. a, Simulation and experimental results for characterizing the non-separability of superposition state 3. **b,** Simulation and experimental results for characterizing the non-separability of superposition state 4.

Supplementary Note 7. Common-path self-reference scheme for the proposed off-axis digital holography technique

Here we carry out an additional experiment to demonstrate a common-path self-reference scheme for our proposed off-axis digital holography-enabled non-separability measurement technique. Displayed in Fig. S6a is the experimental setup. As illustrated in Fig. S6a, generated vectorial structured light is split to two parts by a beam splitter (BS), namely, the signal arm and the reference arm. The reference arm is received by a collimator (Col. 1). The Col.1 is connected to a single mode fiber (SMF). Although the SMF doesn't support the higher-order vectorial structured light, it is capable of receiving part of the vectorial structured light. In this case, the SMF acts as a spatial filter which creates self-reference reference beam, at the cost of high power loss. The SMF is then connected to a Col. 2 to convert the fiber-guided Gaussian beam to a free-space Gaussian beam. A polarizer controls its polarization to 45°.

A beam expander (BE) expands the beam to obtain the satisfied reference beam for off-axis digital holography. For the signal beam, a neutral density filter (NDF) is applied to balance the power ratio of two arms to ensure the high-contrast of off-axis holograms. A charge-coupled device (CCD) detects the two-channel off-axis holograms to characterize the non-separability of the vectorial structured light.

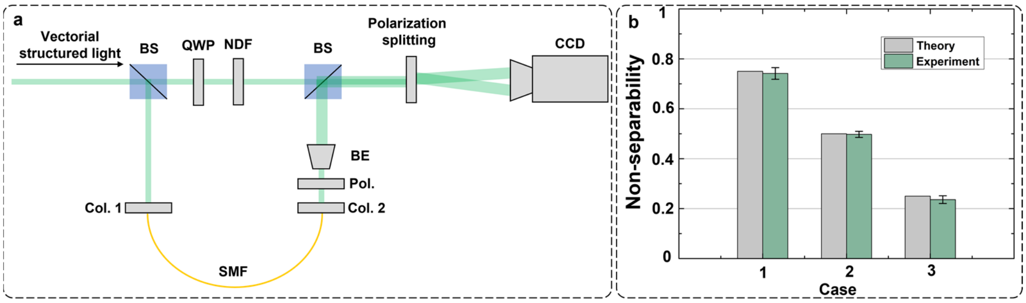

Fig. S6. Additional experiment to demonstrate the proposed common-path self-reference holography scheme. a, Experimental setup for the common-path self-reference holography scheme. b, Experimental results for characterizing the non-separability of vectorial structured light based on the common-path self-reference holography scheme. BS, beam splitter; Col., collimator; SMF, single mode fiber; Pol., polarizer; BE, beam expander; CCD, charge-coupled device; QWP, quarter wave plate; NDF, neutral density filter.

Here we experimentally demonstrate the non-separability measurement of three vectorial structured light beams with l=2. Such three vectorial structured light beams feature different non-separability of 0.75, 0.5 and 0.25. Figure S6b showcases the experimental results for non-separability measurement of these three vectorial structured light beams based on the common-path self-reference scheme. For each vectorial structured light beam, it is measured for 50 times to calculate the measurement standard deviation to represent the error margins. Experimental results are all in good agreement with theoretical ones.

Supplementary Note 8. Details and additional results for charactering the non-separability contributions of a four-mode-superposition state

In this section, we show the details for characterizing the non-separability contributions of a four-mode superposition state. Such a superposition state can be written as,

$$E_{sup}=\sum_{l}^{1,-3,5,-7}[A_l E_{OV}(l)\mathbf{e}_{\mathrm{L}}+B_l E_{OV}(-l)\mathbf{e}_{\mathrm{R}}] \tag{s16}$$

where $E_{OV}(l)$ is the l-th optical vortex mode and A_l corresponds to its amplitude coefficient, $E_{OV}(-l)$ is the -l-th optical vortex mode and B_l corresponds to its amplitude coefficient, $\mathbf{e}_{\mathrm{L}}$ and $\mathbf{e}_{\mathrm{R}}$ denote the left circularly polarized state and right circularly polarized state, respectively. We set

A_1=A_{-3}=A_5=A_{-7}=0.25 , B_1=0.12 , B_{-3}=0.75 , B_5=0.09 and B_{-7}=0.04 . Thus, the theoretical non-separability values of the four modes are 0.7802, 0.6, 0.6374, 0.3120, respectively. Here we characterize the non-separability values of such a superposition state by utilizing our technique. Figure S7a illustrates simulation results for characterizing the non-separability contributions of such a four-mode-superposition vectorial state, including simulated off-axis holograms, SF domains and reconstructed intensity profiles and phasefronts. Similarly, Fig. S7b indicates experimentally measured ones.

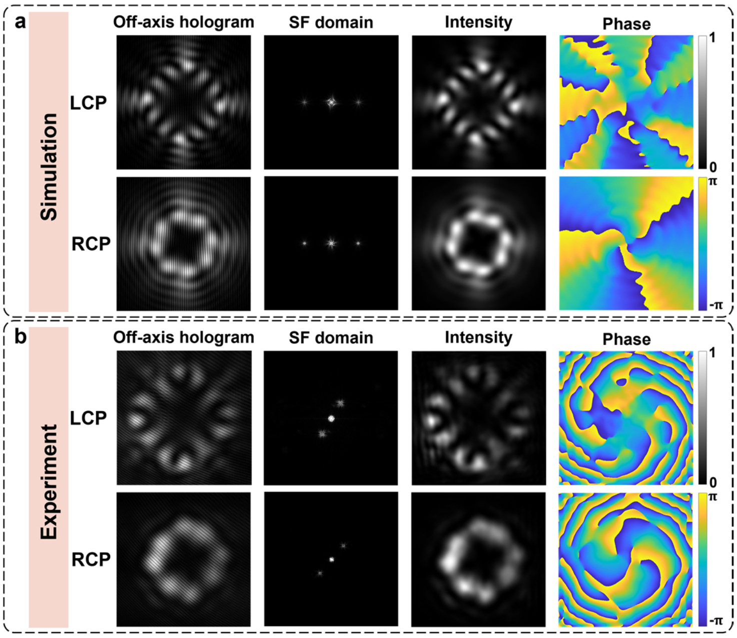

Fig. S7. Additional results for characterizing the non-separability contributions of a four-mode-superposition vectorial state. a, Simulation results for characterizing the non-separability contributions of a four-mode-superposition vectorial state. **b,** Experimental results for characterizing the non-separability contributions of a four-mode-superposition vectorial state.

Supplementary Note 9. Operation condition and limitations of off-axis digital holography

Here we evaluate how the spatial resolution and the off-axis angle of the interference affect the efficiency of off-axis digital holography. Without loss of generality, we assume that two beams only have an off-axis angle in the x direction. Such an x-directional off-axis angle is defined as θ_x. Correspondingly, it leads to a shift in the spatial-frequency domain, and the shift can be calculated as,

$$u_0=\frac{2\pi}{\lambda}\sin(\theta_x) \quad \text{(s17)}$$

where λ is the wavelength. After obtaining the off-axis interferogram, one can obtain the conceptual spatial-frequency domain illustrated in Fig. S8a. The auto-correlation (DC) term is represented as a red

circle, while the cross-correlation (CC) term is in yellow. Assuming that the maximum spatial frequency of the sample beam is ω_s on both axes, each of the CC terms occupies a spatial bandwidth capacity of [-ω_s,ω_s], and the DC terms occupy a spatial bandwidth capacity of [-2ω_s,2ω_s]. In addition, according to the Nyquist–Shannon sampling theorem, the cut off frequency of the spatial-frequency domain is half the sampling frequency. Thus, if the x-directional spatial resolution of utilized detector is Δx, the coinciding cutoff angular frequency is calculated as,

$$\omega_{c,u}=\frac{2\pi}{2\Delta x}=\frac{\pi}{\Delta x} \tag{s18}$$

that is, the maximal frequency of its spatial-frequency domain is $\frac{\pi}{\Delta x}$. Then we consider two extreme cases: i) As showcased in Fig. S8b, if the tilt angle is too large, the CC term will come to the boundary of the spatial-frequency domain. Thus, to avoid such a phenomenon, we have $u_0+\omega_s\leq\omega_{c,u}$. After derivation, we can obtain the maximal tilt angle, $\sin(\theta_x)\leq\frac{\lambda}{2\Delta x}-\frac{\lambda\omega_s}{2\pi}$; ii) As indicated in Fig. S8c, note that the tilt angle is required to be large enough to separate DC term from CC term. In this case, we have $u_0\geq 2\omega_s$, that is, $\sin(\theta_x)\geq\frac{\lambda\omega_s}{\pi}$. In summary, to ensure the efficiency of off-axis digital holography, the tilt angle should satisfy $\frac{\lambda\omega_s}{\pi}\leq\sin(\theta_x)\leq\frac{\lambda}{2\Delta x}-\frac{\lambda\omega_s}{2\pi}$.

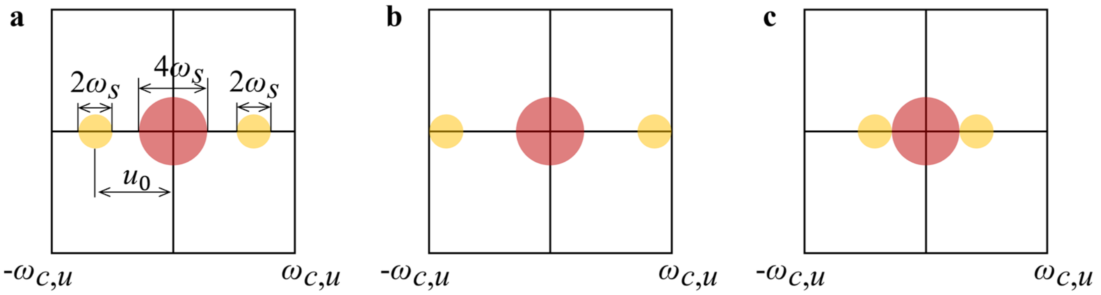

Fig. S8. Conceptual spatial-frequency domain of off-axis interferograms. a, A normal case that the CC term is separated from the DC term, and neither of these two terms exceeds the cutoff frequency. **b**, An extreme case that the CC term locates at the boundary of the spatial-frequency domain. **c**, An extreme case that the CC term is just separated from the DC term.

Supplementary Note 10. Simulation results for propagating a superposition vectorial state through atmospheric turbulence

In this section, we show additional simulation results for propagating a superposition vectorial state through atmospheric turbulence, to evaluate how the non-separability contributions change after passing through media that induce modal crosstalk.

Firstly, we define a multiplex state, written as,

$$E_{\mathrm{Sup}} = [\cos\gamma E_{OV}(l_1) + \sin\phi E_{OV}(-l_2)]\mathbf{e_L} + [\sin\gamma E_{OV}(-l_1) + \cos\phi E_{OV}(l_2)]\mathbf{e_R} \quad \text{(s19)}$$

where E_{Sup} represents the light field of a vectorial structured light superposition state. Such a light field is superimposed by two vectorial structured light beams with mode indices of l_1 and l_2, respectively. We set l_1=4, l_2=2, $\gamma=\frac{\pi}{5}$, and $\phi=\frac{\pi}{10}$. Thus, for mode l_1=4, it features non-separability of about 0.9511. For mode l_2=2, it possesses non-separability of 0.5878. The polarization distribution of such a superposition state is illustrated in Fig. S9a, green symbols represent LCP states while red symbols represent RCP states. Its LCP component and RCP component are also shown in Fig. S9a. Then such an input state propagates through a simulated turbulence phase screen and finally obtains the output state in Fig. S9b. For the output state, it is deteriorated by the atmospheric turbulence, leading to changed non-separability contributions of l_1=4 and l_2=2 (the non-separability contribution of l_1=4 changes to 0.6033 and that of l_2=2 changes to 0.9413). Of course, the overall space-polarization non-separability remains invariance, with an input value of 0.9974 and an output value of 0.9939.

Although the measured non-separability contributions don't keep invariance in media that induce modal crosstalk, they can be utilized in many other scenarios. For instance, for media that only induce geometric phase (e.g., out-plane perturbations of optical fibers), the non-separability contributions of different modal groups can keep invariance. Hence, they can be applied in such situations. Moreover, such non-separability contributions can be applied to quantitively characterize the spin-orbit coupling within specific modal groups. In addition, for optical communication or optical sensing systems based on multi-mode fibers (MMFs), intra-modal-group crosstalk is much more likely to occur than inter-modal-group crosstalk (since the effective refractive index differences within the mode group is much smaller). In this case, our technique may be useful to provide more information within specific modal groups. For example, a measured non-separability contribution of 1 represents the worst situation, i.e., crosstalk of the input pure mode within the mode group is completely random.

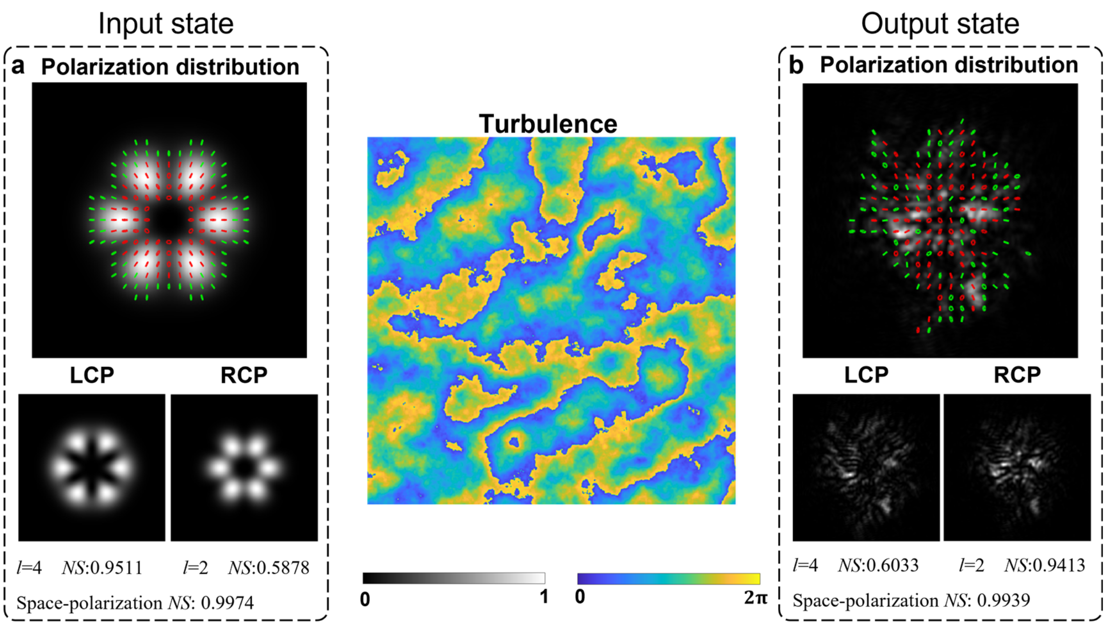

Fig. S9. Simulation results for propagating a superposition vectorial state through atmospheric turbulence. a, Simulation results for the input state, including its polarization distribution, LCP component and RCP component. **b,** Simulation results for the output state, including its polarization distribution, LCP component and RCP component. NS, non-separability.

Supplementary Note 11. Theoretical proof and simulation results which demonstrate that Stokes parameters can't be utilized to accomplish the digital modal decomposition process

In this section, we showcase that even the full Stokes parameters of a vectorial structured light beam are provided, one can't perform digital decomposition on it, since such a vectorial structured light beam may be thought to be superposed by different pairs of beams.

Without loss of generality, we consider a specific vectorial light field superposed by two circularly polarized components. The two circularly polarized components feature the same amplitude distribution but different phasefronts. Thus, such a vectorial structured light beam can be expressed as,

$$\begin{bmatrix}1\\ i\end{bmatrix}\cdot|E(x,y)|\cdot e^{i\varphi_1(x,y)}+\begin{bmatrix}1\\ -i\end{bmatrix}\cdot|E(x,y)|\cdot e^{i\varphi_2(x,y)} \quad \text{(s20)}$$

where $\begin{bmatrix}1\\ i\end{bmatrix}$ and $\begin{bmatrix}1\\ -i\end{bmatrix}$ are respectively the Jones matrices of left circular polarization and right circular polarization, $|E(x,y)|$ is the amplitude distribution, $\varphi_1(x,y)$ and $\varphi_2(x,y)$ are the phasefronts of these two polarization components. The polarization distribution of such a vectorial structured light beam can be defined by full Stokes parameters $S_0(x,y)$, $S_1(x,y)$, $S_2(x,y)$, and $S_3(x,y)$. Such four Stokes parameters can be obtained by measuring four Stokes intensities, namely, $I_H(x,y)$, $I_D(x,y)$, $I_L(x,y)$, and $I_R(x,y)$,

corresponding to the linearly polarized horizontal (H), diagonal (D) and circular-left (L) and circular-right (R) polarizations. The full Stokes parameters can be calculated as,

$$\begin{cases} S_0(x,y)=I_L(x,y)+I_R(x,y) \\ S_1(x,y)=2I_H(x,y)\text{-}S_0(x,y) \\ S_1(x,y)=2I_D(x,y)\text{-}S_0(x,y) \\ S_3(x,y)=I_R(x,y)\text{-}I_L(x,y) \end{cases} \tag{s21}$$

that is, the polarization distribution of a vectorial structured light beam can be specifically determined by $I_H(x,y)$, $I_D(x,y)$, $I_L(x,y)$, and $I_R(x,y)$. Here we calculate $I_H(x,y)$, $I_D(x,y)$, $I_L(x,y)$, and $I_R(x,y)$ of the mentioned vectorial structured light:

$$\begin{aligned} I_H(x,y)&=E_H(x,y)\cdot E_H(x,y)^* \\ &=[|E(x,y)|\cdot e^{i\varphi_1(x,y)}+|E(x,y)|\cdot e^{i\varphi_2(x,y)}]\cdot[|E(x,y)|\cdot e^{i\varphi_1(x,y)}+|E(x,y)|\cdot e^{i\varphi_2(x,y)}]^* \\ &=2|E(x,y)|^2+2|E(x,y)|^2\cos[\varphi_1(x,y)\text{-}\varphi_2(x,y)] \end{aligned} \tag{s22}$$

Similarly,

$$I_D(x,y)= 2|E(x,y)|^2-2|E(x,y)|^2\sin[\varphi_1(x,y)\text{-}\varphi_2(x,y)] \tag{s23}$$

$$I_L(x,y)=|E(x,y)|^2 \tag{s24}$$

$$I_R(x,y)=|E(x,y)|^2 \tag{s25}$$

Considering the superposition of another two light fields, written as,

$$\begin{bmatrix}1\\ i\end{bmatrix}\cdot|E(x,y)|+\begin{bmatrix}1\\ \text{-}i\end{bmatrix}\cdot|E(x,y)|\cdot e^{i[\varphi_2(x,y)\text{-}\varphi_1(x,y)]} \tag{s26}$$

Such a vectorial structured light beam is synthesized by a circular-left component $|E(x,y)|$ and a circular-right component $|E(x,y)|\cdot e^{i[\varphi_2(x,y)\text{-}\varphi_1(x,y)]}$. Here we also evaluate its four Stokes intensities, calculated as,

$$I'_H(x,y)=\{|E(x,y)|+|E(x,y)|\cdot e^{i[\varphi_2(x,y)\text{-}\varphi_1(x,y)]}\}\cdot\{|E(x,y)|+|E(x,y)|\cdot e^{i[\varphi_2(x,y)\text{-}\varphi_1(x,y)]}\}^*$$

$$=2|E(x,y)|^2+2|E(x,y)|^2\cos[\varphi_1(x,y)\text{-}\varphi_2(x,y)] \tag{s27}$$

$$I'_D(x,y)=2|E(x,y)|^2-2|E(x,y)|^2\sin[\varphi_1(x,y)\text{-}\varphi_2(x,y)] \tag{s28}$$

$$I'_L(x,y)=|E(x,y)|^2 \tag{s29}$$

$$I'_R(x,y)=|E(x,y)|^2 \tag{s30}$$

It can be seen that the mentioned two vectorial structured light beams have the same Stokes parameters but different synthesized components. Thus, although the full Stokes parameters of a vectorial structured light beam are measured, one can still not decompose it into two orthogonal components for modal tomography.

Here, we also carry out a simulation to demonstrate our conclusion. For perfect OV beams, they possess the same beam size (i.e., radius and ring width) at different mode orders (i.e., topological charges). Such a perfect OV beam can be defined as,

$$E_{perfect}(r,\theta)=\exp[-\frac{(r\text{-}r_0)^2}{\Delta r^2}]e^{il\theta} \tag{s31}$$

where r and θ are polar coordinates, r_0 and Δr define the radius of the annulus and its width. As illustrated in Fig. S10a, we show the simulated superposition of two perfect OV beams: one is a left-circular OV beam with l=1, and the other is a right-circular OV beam with l=-1. Their superposition results in the radial vector beam indicated at the top of Fig. S10. Here we use red circles and green circles to represent left-circular polarizations and right-circular polarizations, respectively. However, as displayed in Figs. S10 b and c, such a radial vector beam can be obtained through other kinds of superpositions.

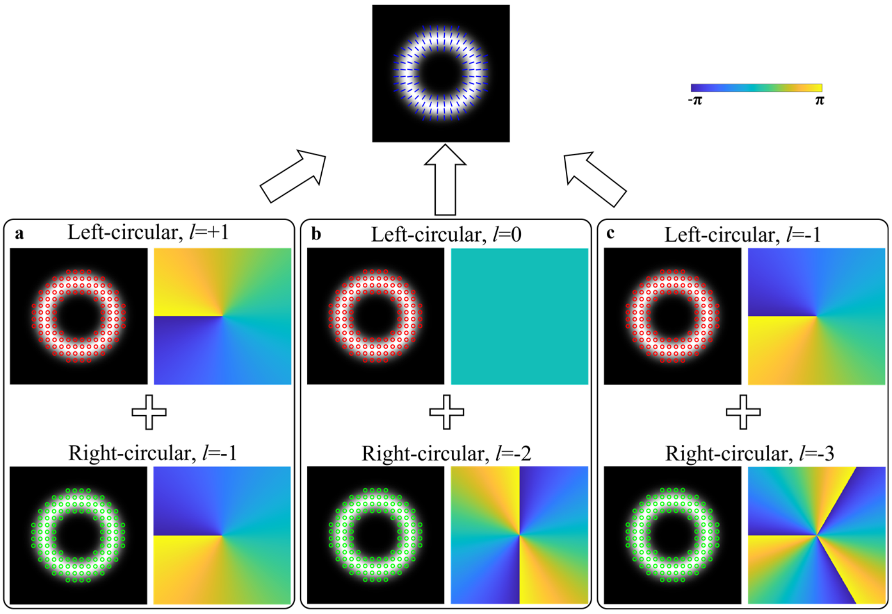

Fig. S10. Simulation results for superposing perfect OV beams to obtain the same radial vector beam. a, Illustration of two perfect OV beams: a left-circular OV beam with l=1 and a right-circular OV beam with l=-1. **b,** Illustration of two perfect OV beams: a left-circular OV beam with l=0 and a right-circular OV beam with l=-2. **c,** Illustration of two perfect OV beams: a left-circular OV beam with l=-1 and a right-circular OV beam with l=-3.

To conclude, one can't obtain the full modal information of a vectorial structured light beam through Stokes tomography or polarization sensitive cameras. However, holography enables not only achieving measuring the polarization distribution of a vectorial structured light beam but also retrieving its modal information in orthogonal polarization channels.

Supplementary References

1. Beijersbergen, M. W., Allen, L., van der Veen, H. E. L. O. & Woerdman, J. P. Astigmatic laser mode converters and transfer of orbital angular momentum. *Opt. Commun.* **96**, 123-132 (1993).
2. Yao, A. M. & Padgett, M. J. Orbital angular momentum: origins, behavior and applications. *Adv. Opt. Photonics*. **3**, 161-204 (2011).
3. Berry, H. G., Gabrielse, G. & Livingston, A. E. Measurement of the Stokes parameters of light. *Appl. Optics*. **16**, 3200-3205 (1977).
4. Sztul, H. I., Nolan, D. A., Alfano, R. R. & Milione, G. Higher-Order Poincaré Sphere, Stokes Parameters, and the Angular Momentum of Light. *Phys. Rev. Lett.* **107**, 53601 (2011).
5. Liang, Y. et al. Reconfigurable structured light generation and its coupling to air–core fiber. *Advanced Photonics Nexus*. **2**, 36015 (2023).
